# Exploring organic photosensitizers based on hemicyanine derivatives: a sustainable approach for preparation of amide linkages†

**DOI:** 10.1039/c8ra06232c

**Published:** 2018-09-05

**Authors:** Harnimarta Deol, Manoj Kumar, Vandana Bhalla

**Affiliations:** Department of Chemistry, UGC Sponsored Centre for Advanced Studies-II, Guru Nanak Dev University Amritsar 143005 Punjab India vanmanan@yahoo.co.in

## Abstract

Hemicyanine derivatives C1–C4 have been synthesized and show strong absorption in the visible region, good water solubility, efficient intersystem crossing, a high singlet oxygen quantum yield and high ability to transport electrons from the donor to acceptor. These hemicyanine derivatives were utilized as photocatalysts in additive/base free oxidative amidation of aromatic aldehydes in mixed aqueous media under visible light irradiation at low catalytic loading. The hemicyanine derivative C4 exhibited recyclability upto four cycles and reusability upto five cycles in oxidative amidation of aromatic aldehydes. Among all the hemicyanine derivatives, C4 shows a high photocatalytic efficiency due to a high singlet oxygen quantum yield. All the mechanism investigations showed involvement of reactive oxygen species generated by the organic triplet photosensitizer based on hemicyanine derivative for carrying out oxidative amidation of aldehyde. Our results will encourage the design of new “metal free” organic photosensitizers and their application in photocatalysis.

## Abstract 2

Hemicyanine derivatives C1–C4 have been synthesized and utilized as photocatalysts in additive/base free oxidative amidation of aromatic aldehydes in mixed aqueous media under visible light irradiation at low catalytic loading.

## Graphical abstract

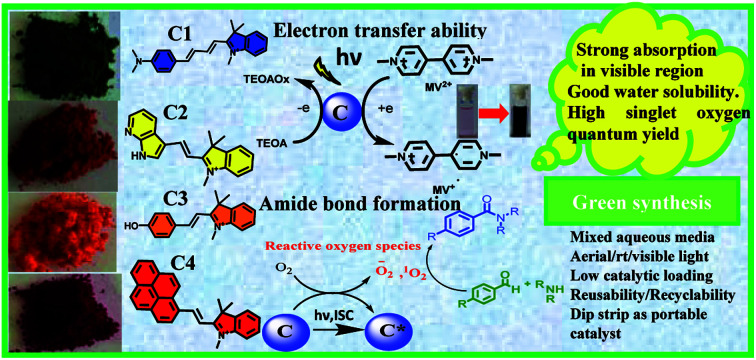

## Introduction

**Scheme 1:**
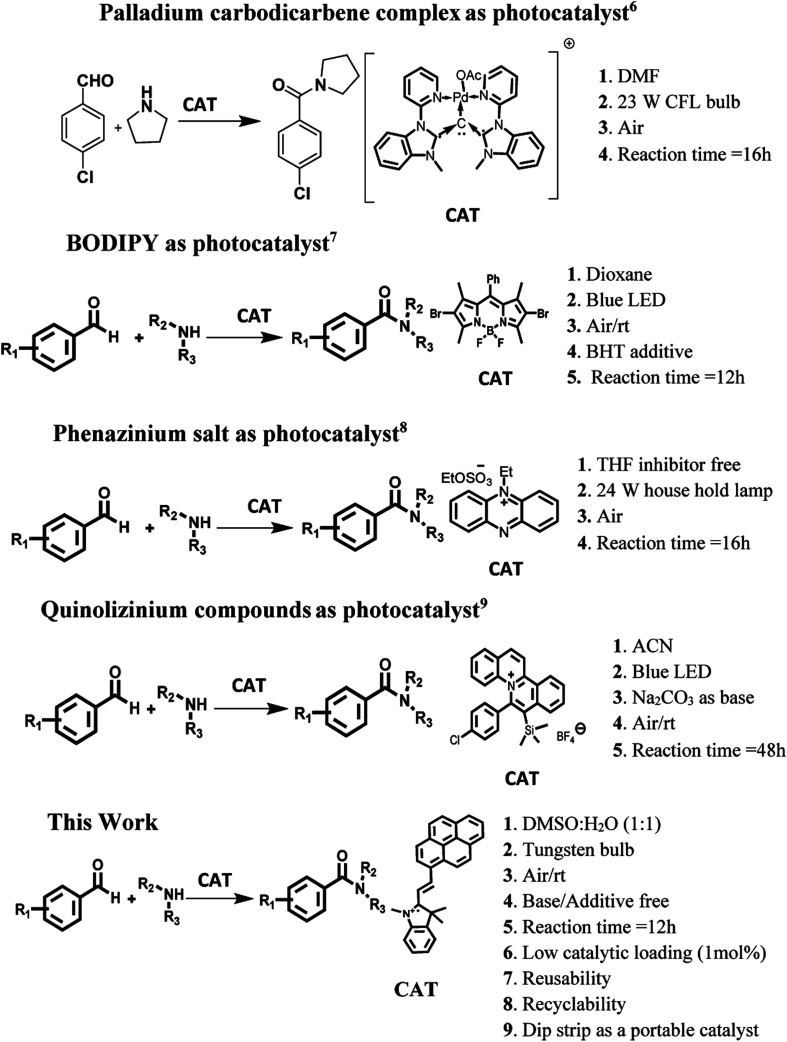
Oxidative amidation of aromatic aldehyde.

The amide linkage is a structural backbone of proteins and peptides. These linkages are also predominant in medicinally important compounds, natural products and materials having industrial applications. The reason behind the high prevalence of the amide linkage is its high stability, polarity and conformational diversity.^1^ Conventional protocols for the preparation of amide bonds generate a significant amount of chemical waste/side products and hence have poor atom economy.^2^ Thus, a lot of efforts are being devoted to making the preparation of the amide linkage more ‘green’. One of the best approache to achieve the targets of green synthesis is to carry out the reaction in the presence of some catalytic species. The catalysts are special as they influence the kinetics of the reaction positively, provide an alternative pathway to the reaction, and themselves remain unchanged. To date, a variety of reaction protocols centred around various catalytic systems based on transition metals^2,3^ and N-heterocyclic carbenes^4^ have been reported for amide bond formation. The majority of these catalyzed synthetic protocols require the presence of an oxidant, strong thermal conditions and an inert atmosphere which in fact reduces the economic and environmental advantages of the strategy. These limitations have been overcome by utilization of “photosensitizers” as the photocatalysts for the preparation of the amide bond *via* oxidative amidation of aldehydes. In this context, a variety of commercially available ruthenium/iridium metal based complexes^1,5^ and dyes have been utilized as photocatalysts. Very recently, palladium carbodicarbene complex^6^ has been utilized as photocatalyst for the construction of amide bond in organic reaction media under visible irradiation. The main limitations of this approach are the requirement of the toxic, costly and synthetically difficult organometallic complexes as the catalytic species. Further, high loading of these photocatalysts are essential in the reaction mixture to get the target compound in good yield. The reason behind necessity of high loading of photocatalyst is weak/moderate absorption of these materials in visible region. Unfortunately, structural modification of these photocatalysts to enhance their absorption in visible region is also difficult. To overcome these limitations, several organic triplet photosensitizers based on BODIPY,^7^ phenazinium^8^ and quinolizinium compounds^9^ scaffold have been synthesized and tested for the photocatalytic synthesis of amide bonds (Scheme 1). In comparison to ruthenium/iridium heavy metal based complexes, these organic triplet photosensitizers absorb strongly in visible region and have long life time of the triplet excited state. Most of these photocatalytic systems are themselves capable of activating O_2_ under light irradiation and hence external oxidant is not needed. However, the economic/environmental advantages of these approaches are decreased due to their multistep synthetic routes, tedious purification and also the presence of boron as heavy atom (in BODIPY based catalytic system). Further, most of these systems work only in organic media and in some cases the desired product is furnished in decent yield only in the presence of blue LED as source of radiation which again decreases the economic and environmental advantages of the strategy. Thus, development of an efficient photocatalyst/organic triplet photosensitizer which could work efficiently under visible light irradiation in base/additive free conditions in aqueous media is still a challenge.

Our research work involves the development of new photocatalytic systems for harvesting solar radiations for pursuing various organic transformations.^10^ In continuation of our efforts in this direction, we were then interested to develop a dye based photocatalytic system for amide bond formation *via* oxidative amidation of aromatic aldehydes in aqueous media under eco-friendly conditions. For preparation of such photocatalytic system, hemicyanine dye is scaffold of our choice due to its strong absorption in visible region, good water compatibility, straightforward synthetic route, ease of modification, long lived triplet excited state and its ability to generate reactive oxygen species. With these unique photophysical and photochemical properties hemicyanine dyes have multidisciplinary applications in various fields such as photodynamic therapy (PDT), bioimaging, construction of dye sensitized organic solar cells and pharmaceutical industry *etc.*^11^

Recently, from our lab we reported a hemicyanine derivative C1 which emits in NIR region and showed selective and sensitive response towards HSA in aqueous media.^12^ As a test of our hypothesis, we planned to the examine the photocatalytic efficiency of NIR active hemicyanine derivative C1 in additive/base free oxidative amidation of aromatic aldehydes in mixed aqueous media under visible light irradiation. To our delight, the reaction worked well and the desired product was obtained in 72% yield (*vide infra*). Encouraged by this result, we plan to synthesized a series of photocatalysts (C1–C4) based on hemicyanine dye and examine their efficiency in the oxidative amidation reaction. These photocatalysts based on hemicyanine dye were prepared by simple condensation of various aldehydes with 1,2,3,3-tetramethyl-3*H*-indol-1-ium in ethanol. The photocatalytic activity of these hemicyanine derivatives were examined in additive/base free oxidative amidation of aromatic aldehydes under aerial conditions in aqueous media using tungsten filament bulb as the irradiation source. Amazingly, among all the synthesized derivatives, C4 having pyrene as donor unit exhibited high efficiency and furnished the target compound in good yield. The hemicyanine derivative C4 exhibited recyclability upto four cycles and reusability upto five cycles in oxidative amidation of aromatic aldehyde reaction. The high efficiency of derivative C4 may be attributed to its strong absorption in visible region, good water solubility, high singlet oxygen quantum yield (*Φ*_Δ_ = 0.85) and efficient intersystem crossing. The work being presented in this manuscript demonstrates the utilization of hemicyanine derivatives as efficient photocatalysts for oxidative amidation of aldehydes under solar radiation and under solvent free conditions. Unprecedented, this is the first report regarding utilization of hemicyanine derivatives as organic photosensitizer in photocatalysis under mild condition (aqueous media, base/additive free visible light and aerial conditions, low catalytic loading). Further, the ‘dip strip’ as portable catalytic system was prepared by dipping filter paper strip into the solution of derivative C4 for carrying out oxidative amidation of aromatic aldehyde under visible light irradiation.

## Results and discussion

### Synthesis of the photosensitizers based on hemicyanine derivatives

**Scheme 2:**
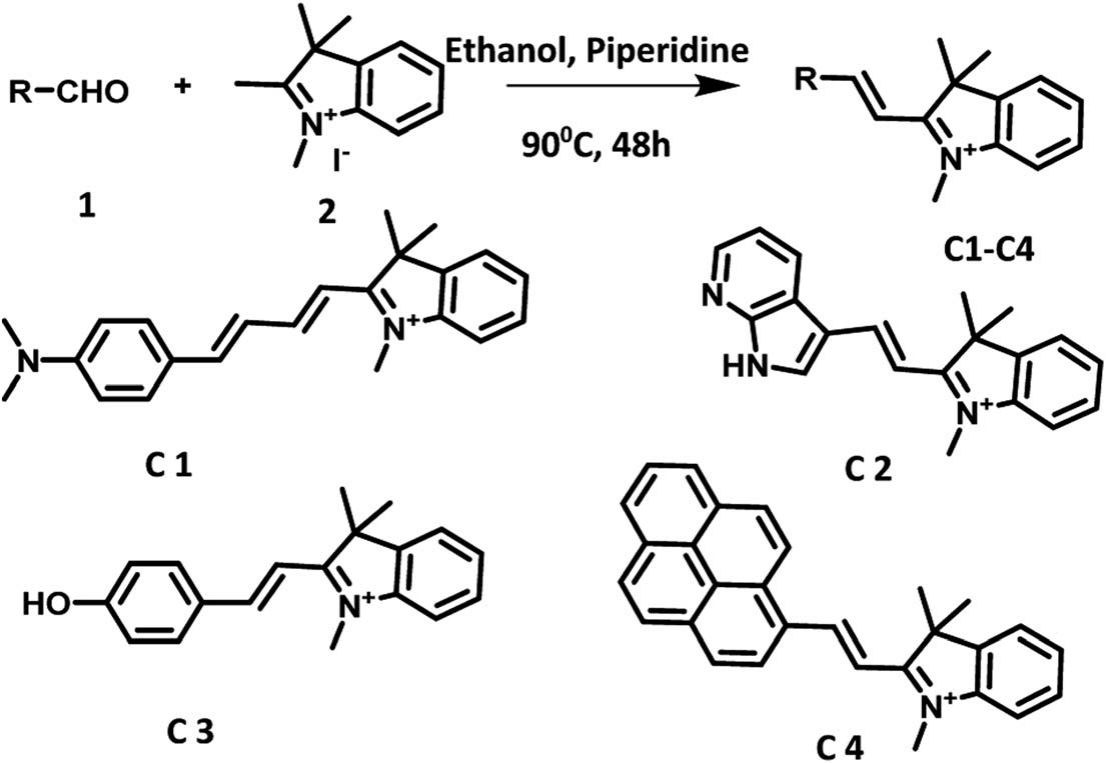
Synthesis of photocatalysts based on hemicyanine derivatives (C1–C4).

Derivatives C1, C3 and C4 were synthesized by following the procedure reported in literature (Fig. S40–S44 in ESI†).^12–14^ Derivative C2 was synthesized by condensation of 1,2,3,3-tetramethyl-3*H*-indol-1-ium with 2,7-azaindole-3-carboxaldehyde in ethanol (Scheme 2).^15^ The structure of C2 was characterized by various spectroscopic and analytical studies (Fig. S37–S39 in ESI†).

### Photophysical properties

The UV-vis and fluorescence studies of derivatives C1–C4 were examined in DMSO : H_2_O (1 : 1). The results were summarized in Table 1 which clearly show that derivatives C1, C3 and C4 exhibit absorption/emission at longer wavelength in comparison to that of derivative C2. The comparatively red shifted emission in case of derivatives C1, C3 and C4 may be attributed to the presence of relatively stronger donor units and extended conjugation which strongly facilitates the intramolecular charge transfer in these systems (Fig. S1 and S2 in ESI†). Further, the singlet state lifetime of the derivatives C1–C4 were evaluated using the time resolved fluorescence spectroscopy (Fig. S3–S6 in ESI†). In all the derivatives, life time of singlet state was found to be less than 1 ns, thus, long lifetime of their respective triplet state is expected. Derivatives C2 and C3 showed decent quantum yields but in case of derivatives C1 and C4 the low quantum yields were determined (Table 1). The low quantum yields in case of derivatives C1 and C4 further indicate efficient intersystem crossing (ISC) form singlet to triplet excited state in these systems, as a result of which a highly stable/long lived triplet excited state is expected in these derivatives. Further we calculated the singlet oxygen quantum yields for derivatives C1 and C4 using 1,3-diphenylisobenzofuran (DPBF) a known ^1^O_2_ quencher (Fig. S7 and S8 in ESI†).^16^ Both the derivatives showed good efficiency for generation of singlet oxygen (Table 1).

**Table 1:** Photophysical parameters of photosensitizers based on hemicyanine derivatives^a^

Photosensitizer	*λ*_abs_ (nm)	*ε*	*λ*_em_ (nm)	*Φ*_F_	*τ*_avg_ (ns)	*Φ*_Δ_
C1	587	33 000	686	0.04	0.78	0.70
C2	457	25 000	511	0.25	0.85	a
C3	540	60 000	557	0.20	0.8	a
C4	500	32 000	560/610	0.03	0.50	0.85

a *ε* = molar extinction coefficient M^−1^ cm^−1^, *Φ*_F_ = fluorescence quantum yield, *τ*_avg_ = average lifetime of singlet state, *Φ*_Δ_ = singlet oxygen quantum yield, a = could not be detected.

Further, we checked the photostability of hemicyanine derivatives C1–C4 by irradiating their oxygen saturated solutions in mixed aqueous media for 36 h (Fig. S9–S12 in ESI†). The derivatives C1–C3 showed good photostability even after continuous irradiation for 36 h. However, in case of a derivative C4, 30% decrease in absorbance intensity was observed after 36 h.

**Fig. 1:**
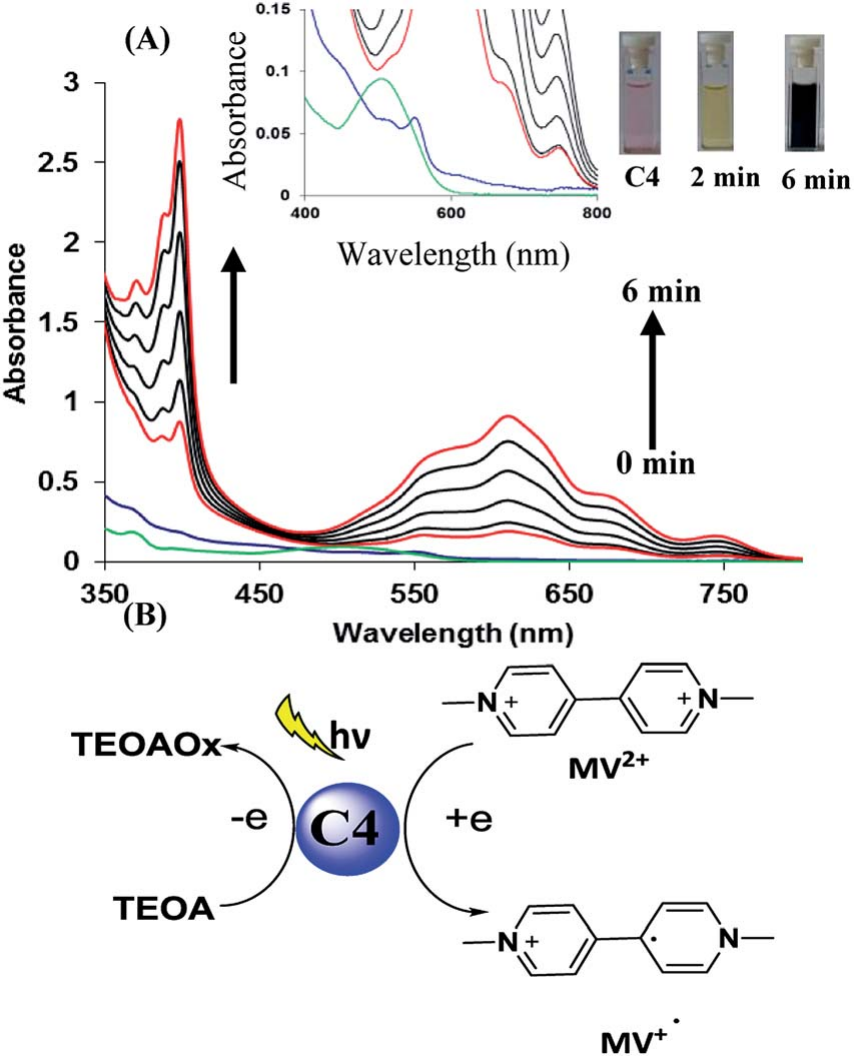
(A) Absorption spectral changes of derivative C4 (0.02 mM) in presence of MV^2+^ (0.2 mM) and TEOA (50 mM) in DMSO under room light and inert atmosphere. Inset: absorption spectral changes upon addition of TEOA in solution of derivative C4 and photograph of solution before and after 6 min. (B) Derivative C4 acts mediator and transport the electron from sacrificial donor to acceptor under visible light.

In the next part of our work, we planned to examine the potential of photosensitizer based on hemicyanine derivative to transport electron from donor to acceptor unit which is key step in photocatalysis. For this we chose three component system consisting of methyl viologen (MV^2+^) as electron acceptor, triethanolamine (TEOA) as sacrificial donor and hemicyanine derivative as the photosensitizer. We observed that upon addition of TEOA in solution of derivative C4, the band at 500 nm due to derivative C4 rapidly decreased along with red shift of 50 nm. These observations indicate a strong interaction between sacrificial donor and derivative C4. Afterwards, we added methyl viologen (MV^2+^) and upon exposure to room light under inert atmosphere within 2 min colour of solution changed from light pink to green. Further, within next 6 min colour of solution changed to dark blue. The whole event was monitored by UV-vis spectroscopy. The UV-vis spectra showed presence of two new bands at *λ* = 395 and 603 nm which gradually increased upon exposure to room light (Fig. 1A). These two bands correspond to the reduced species MV˙^+^ of methyl viologen. On the basis of these results, we proposed that derivative C4* reductively quenched by sacrificial donor and to form C4*^−^, then C4*^−^ transports electron to MV^2+^ and reduced it (Fig. 1B).^17^ We also performed the same experiment with other hemicyanine derivatives (C1–C3), the colour of solution gradually changed from green to blue (Fig. S13–S15 in ESI†). All above results showed that hemicyanine derivatives work as photosensitizers which transport the electron from sacrificial donor to acceptor under visible light.

### Photocatalytic amidation of aromatic aldehyde

After examining the photophysical properties of derivatives C1–C4, our initial investigation focused on the direct oxidative amidation of aromatic aldehyde under visible light irradiation. The reaction between 4-nitrobenzaldehyde (1a, 1.0 equiv.) and pyrrolidine (2a, 3.0 equiv.) in DMSO : H_2_O solvent mixture under the irradiation of tungsten filament bulb at room temperature was chosen as the model reaction. Among all the derivatives, we chose derivative C4 as a photocatalyst for carrying out model reaction due to its high singlet oxygen quantum yield and high efficiency to transport the electron from donor to acceptor. To our delight in presence of 1 mol% of derivative C4, the reaction was completed in 12 h and the desired product was obtained in 82% yield (Table 2, entry 1). Next, we screened different solvents such as THF, MeCN, DMF, dioxane, DMSO and water (Table 2, entries 2–7) as the reaction media for the transformation. The desired product was obtained in comparable yields in case of DMSO and DMSO : H_2_O mixture and much lower yield of product was obtained in water. We chose DMSO : H_2_O (1 : 1) as the reaction media for carrying out further transformations. Further, we also examined the influence of the presence/absence of base in the oxidative amidation of aromatic aldehyde under visible light irradiation (Table 2, entry 8). The presence or absence of base did not influence the yield of the desired product. Further, in the absence of a hemicyanine based photosensitizer or in absence of visible light source, the reaction did not proceed (Table 2, entries 9 and 10). The desired product was obtained in 10% yield when model reaction was setup under inert atmosphere (Table 2, entry 11). This result emphasized that aerial conditions promote the oxidative amidation of aromatic aldehyde under visible light irradiation in presence of photocatalyst based on hemicyanine derivative. Further, we examined the effect of increase in the catalytic loading on the reaction kinetics and efficiency of the reaction. The desired product was obtained in lower yield (60%) (Table 2, entry 12) upon increasing the catalytic loading from 1 mol% to 4 mol%, which may be attributed to the increase in the colour intensity of the solution that hindered the passage of radiations.^8^ We also examined the effect of the presence of external oxidant such as H_2_O_2_ on the reaction kinetics. As expected, the reaction was accelerated and the desired product was furnished in 88% yield after 8 h (Table 2, entry 13). Further, we also checked the effect of BHT as additive in the oxidative amidation of aldehyde under visible light irradiation. However, no significant effect on the yield and rate of reaction was observed (Table 2, entry 14).

**Table 2:** Optimization of reaction conditions

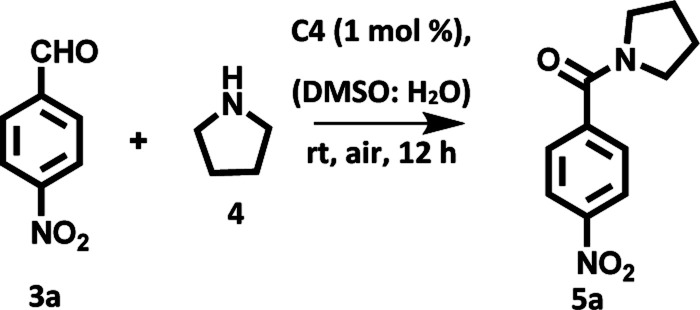					
Entry	Photosensitizer	Additive	Solvent	Time	Yield
1	C4	—	DMSO : H_2_O (1 : 1)	12 h	82%
2	C4	—	DMSO	12 h	80%
3	C4	—	DMF	12 h	15%
4	C4	—	Dioxane		30%
5	C4	—	THF	12 h	55%
6	C4	—	MeCN	12 h	60%
7	C4	—	Water	12 h	30%
8	C4	K_2_CO_3_ as base	DMSO : H_2_O (1 : 1)	12 h	82%
9^a^	—	—	DMSO : H_2_O (1 : 1)	12 h	0%
10^b^	C4	—	DMSO : H_2_O (1 : 1)	12 h	0
11^c^	C4	—	DMSO : H_2_O (1 : 1)	12 h	10%
12^d^	C4	—	DMSO : H_2_O (1 : 1)	12 h	60%
13	C4	H_2_O_2_	DMSO : H_2_O (1 : 1)	12 h	88%
14	C4	BHT	DMSO : H_2_O (1 : 1)	12 h	85%
15	C1	—	DMSO : H_2_O (1 : 1)	20 h	72%
16	C2	—	DMSO : H_2_O (1 : 1)	20 h	50%
17	C3	—	DMSO : H_2_O (1 : 1)	16 h	75%

a No photocatalyst.

b No light.

c Inert atmosphere.

d 4 mol%.

Under these optimized reaction conditions, we also examined the photocatalytic activity of other C1, C2 and C3 hemicyanine derivatives in oxidative amidation of aldehyde. The derivatives C1, C2 furnished the desired product in 72% and 50% yields, respectively after 20 h. On the other hand upon using C3 as the photocatalyst, the desired product was obtained in 75% yield after 16 h (Table 2, entries 15–17). Among all the derivatives C4 showed high photocatalytic activity due to its maximum efficiency for generation of singlet oxygen (*Φ*_Δ_ = 0.85) and high ability to transport the electron as compared to other hemicyanine derivatives. In comparison to the off shelf photosensitizers such as Ru(phen)_3_Cl_2_, Ir(dtbpy)(ppy)_2_PF_6_, Rhodamine B, Methylene blue and Alizarin red S *etc.* (Table S1 in ESI†), the photosensitizers based on hemicyanine derivatives worked well in the oxidative amidation of aldehyde under visible light irradiation.

In order to get more insight into the efficiency of derivative C4, we examined the photophysical properties of derivative C4 in presence of pyrrolidine. The solution of derivative C4 in DMSO showed emission bands at 560/610 nm. Upon addition of 0.1 mmol of pyrrolidine to this solution, the intensity of emission bands at 560/610 nm were quenched completely (Fig. S16 in ESI†). On the other hand, higher equivalents of pyrrolidine were required in case of derivatives C1–C3 (Fig. S17–S19 in ESI†). These studies supported the high efficiency of electron transfer between pyrrolidine and excited state of derivative C4.^7^

**Scheme 3:**
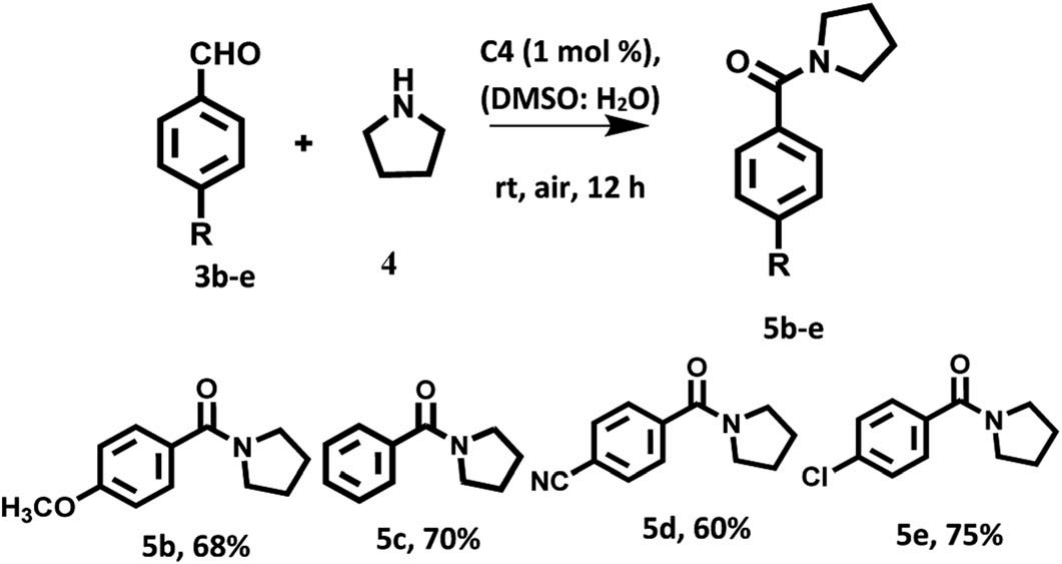
Oxidation amidation of substituted aromatic aldehydes with pyrrolidine using photocatalyst based on hemicyanine derivative C4 under irradiation of visible light at room temperature and aerial conditions.

With the optimized reaction conditions in hand, a variety of aromatic aldehydes were investigated to illustrate the photocatalytic efficiency and scope of the photocatalyst based on hemicyanine derivative C4 under visible light irradiation and aerial conditions. Aromatic aldehydes bearing electron withdrawing groups and electron donating groups furnished the desired products in good yields (Scheme 3).

**Scheme 4:**
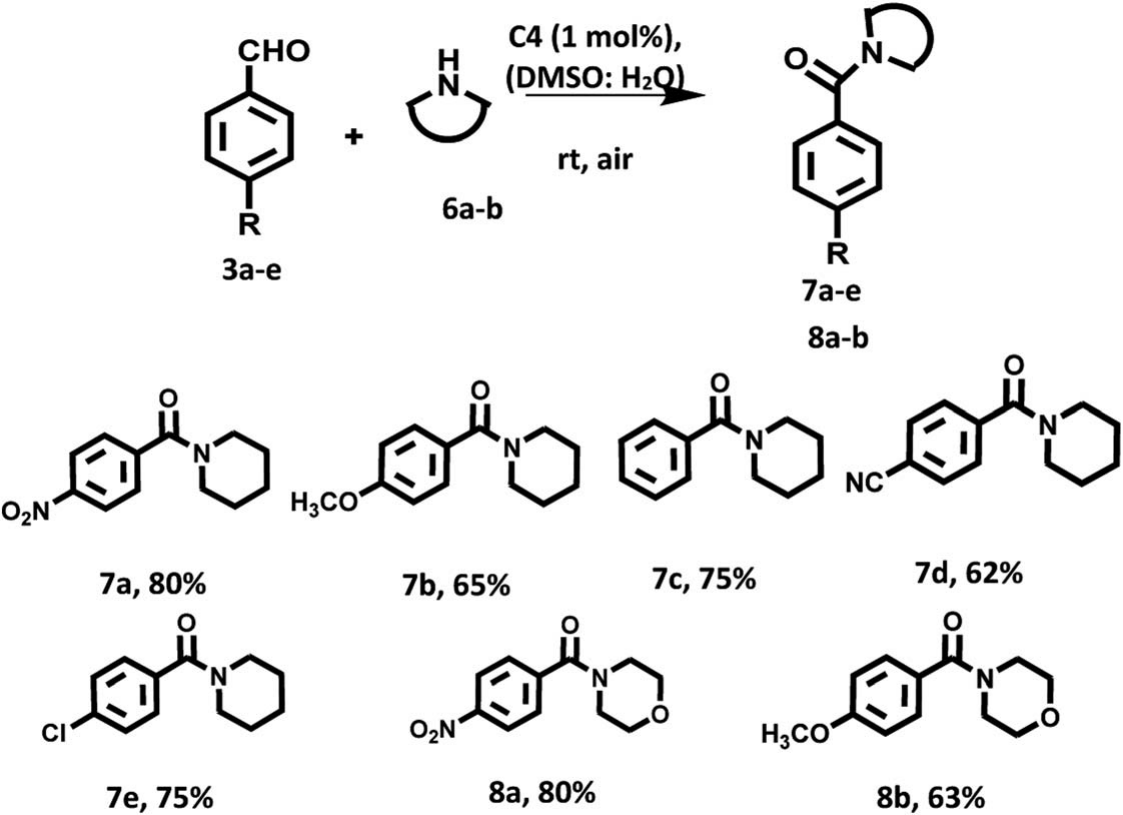
Oxidation amidation of substituted aromatic aldehydes with piperidine/morpholine using photocatalyst based on hemicyanine derivative (C4) under irradiation of visible light at room temperature and aerial conditions.

Furthermore, we examined the substrate scope with respect to amines having six membered rings such as morpholine and piperidine. Amazingly, in presence of these amines as the reaction partner the desired products were obtained in good yields (Scheme 4).

**Scheme 5:**
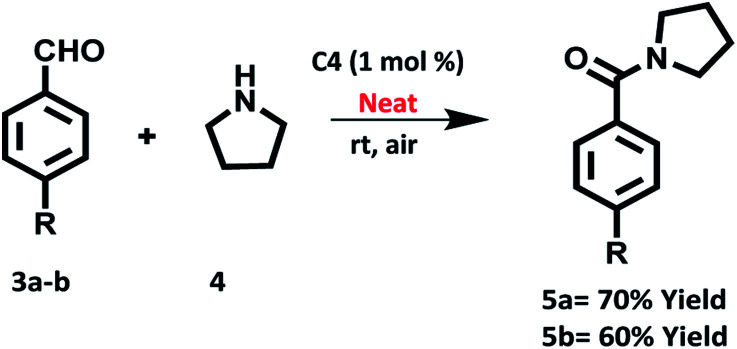
Oxidation amidation of 4-nitrobenzaldehyde with pyrrolidine using photocatalyst based on hemicyanine derivative (C4) under irradiation of visible light at room temperature, neat and aerial conditions.

We also investigated the oxidative amidation of 4-nitrobenzaldehyde (1a, 1.0 equiv.) with pyrrolidine (2a, 3.0 equiv.) under solvent free conditions using of 1.0 mol% of C4 under the irradiation of tungsten filament bulb at room temperature (Scheme 5). To our pleasure, the desired product was obtained in 70% yield after 20 h. On the other hand the reaction between 4-methoxybenzaldehyde (1a, 1.0 equiv.) and pyrrolidine (2a, 3.0 equiv.) under solvent free conditions using 1 mol% of C4 produced the desired product in 60% yield after 36 h. All these studies highlighted the utility of photocatalyst in very mild conditions.

**Scheme 6:**
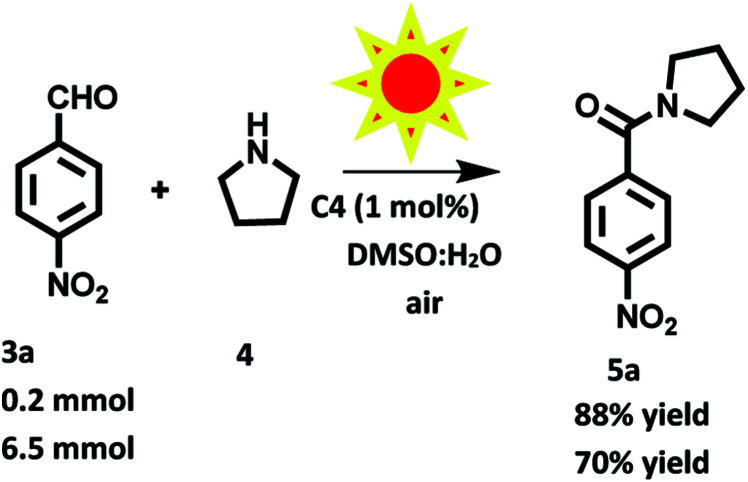
Oxidation amidation of 4-nitrobenzaldehyde with pyrrolidine using photocatalyst based on hemicyanine derivative (C4) under solar radiation at room and aerial conditions.

To further demonstrate the practical application of photocatalyst based on hemicyanine derivative for preparation of amides, we carried out reaction between 4-nitrobenzaldehyde (1a, 1.0 equiv.) and pyrrolidine (2a, 3.0 equiv.) in DMSO : H_2_O (1 : 1) using of C4 (1 mol%) as photosensitizer one at 0.2 mmol and another one at gram scale in presence of solar radiations (Fig. S20 in ESI†). Remarkably, after 8 and 36 h, the desired product was isolated in 88% and 70% yields respectively (Scheme 6). These results showed that C4 found to be more efficient in presence of solar light, which indicate that solar light accelerate reaction rate upto great extent than tungsten bulb using as irradiation source.

**Fig. 2:**
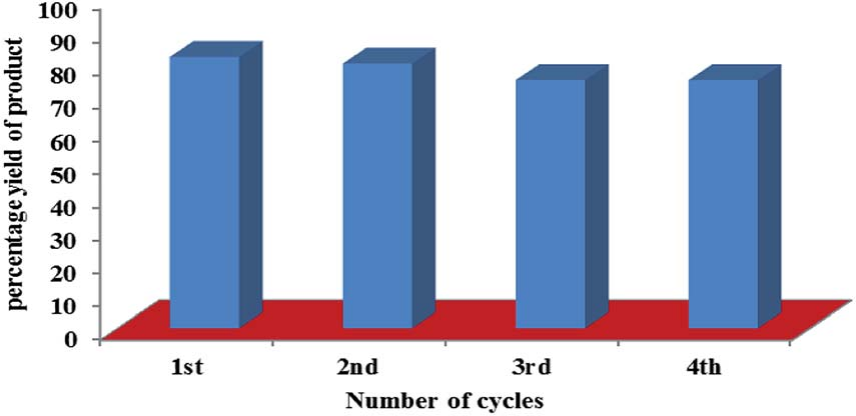
Recyclability of photocatalyst based on hemicyanine derivative (C4) for carrying oxidative amidation of aromatic aldehyde under visible light irradiation.

To determine the recyclability of photocatalytic system, we chose reaction between of 4-nitrobenzaldehyde (1a, 1.0 equiv.) and pyrrolidine (2a, 3.0 equiv.) in DMSO : H_2_O (1 : 1) as the model reaction using 1.0 mol% of derivative C4 as photocatalyst. After completion of the reaction, solvent was evaporated under reduced pressure and the resulting residue was extracted with the ethyl acetate. The organic part was separated and washed with water, dried over Na_2_SO_4_ and concentrated under reduced pressure. The aqueous layer containing photosensitizer was concentrated under reduced pressure and reused for next cycle of oxidative amidation of aldehyde (Fig. S21 in ESI†). The same procedure was followed for four cycles. After the fourth cycle, the yield of the final product was reduced to 62% (Fig. 2).

Further, we examined the reusability of derivative C4 in oxidative amidation of aldehyde. A reusability experiment was carried out using C4 as a catalyst for the oxidative amidation of aldehyde under visible light irradiation. After each cycle, reactants were introduced into the solution of original catalytic system. The derivative C4 was successfully reused upto five cycles and no significant change in the yield of target product was observed (Fig. S22 in ESI†).

**Scheme 7:**
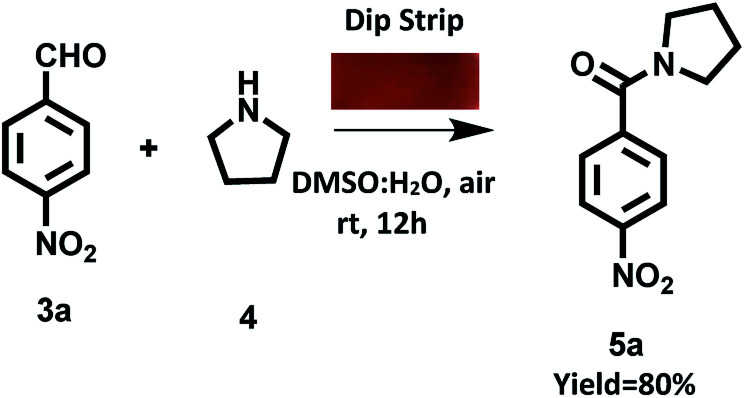
Oxidation amidation of 4-nitrobenzaldehyde with pyrrolidine using dip strip of derivative C4 under visible irradiation at room temperature and aerial conditions.

We also prepared ‘dip strip’ by dip coating a filter paper strip in solution of derivative C4 (Fig. S23 in ESI†). The prepared coated filter paper strip was used as portable catalytic system for carrying out oxidative amidation of aldehyde under visible light irradiation. To our pleasure the reaction was completed in 12 h with 80% yield of target product (Scheme 7).

### Mechanism studies

**Scheme 8:**
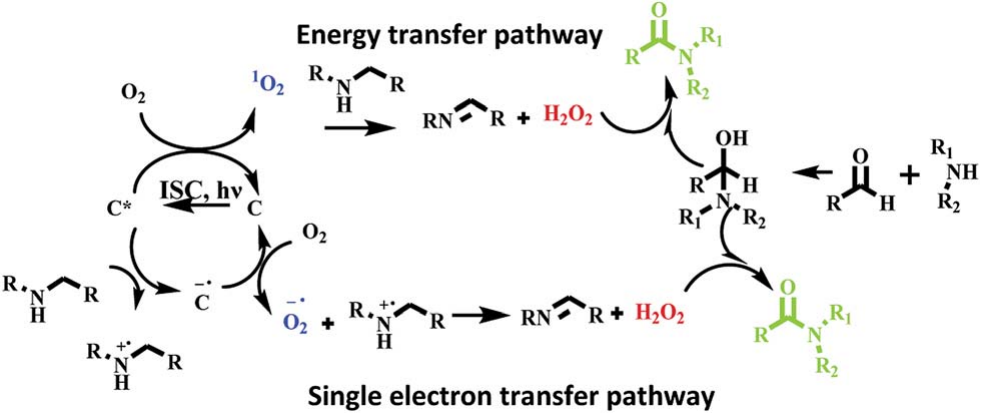
Proposed reaction mechanism for oxidative amidation of aldehyde using photosensitizer C4 under visible light irradiation.

We believe that oxidative amidation of aromatic aldehyde was mediated by reactive oxygen species generated by photosensitizer based on hemicyanine derivative in excited state. For generation of reactive oxygen species, two pathways are suggested. First, energy transfer pathway in which energy of triplet excited state C* transferred to dioxygen and singlet oxygen species was generated, which further reacts with amine to produce H_2_O_2_. The second proposed pathway is activation of dioxygen through a single electron transfer (SET) route to form the superoxide radical O_2_˙^−^ from the radical anion [C]˙^−^, which is generated by SET from the amine to the excited state^3^ [C]*. Then, the active species O_2_˙^−^ produced H_2_O_2_. The *in situ* generated H_2_O_2_ further oxidized α-hydroxy amine to afford the amide (Scheme 8).

To check *in situ* generation of H_2_O_2_, we added KI/CH_3_COOH to the reaction mixture and brown coloration was observed which confirms the presence of H_2_O_2_ in the reaction mixture (Fig. S24 in ESI†).^7,16*c*^

**Scheme 9:**
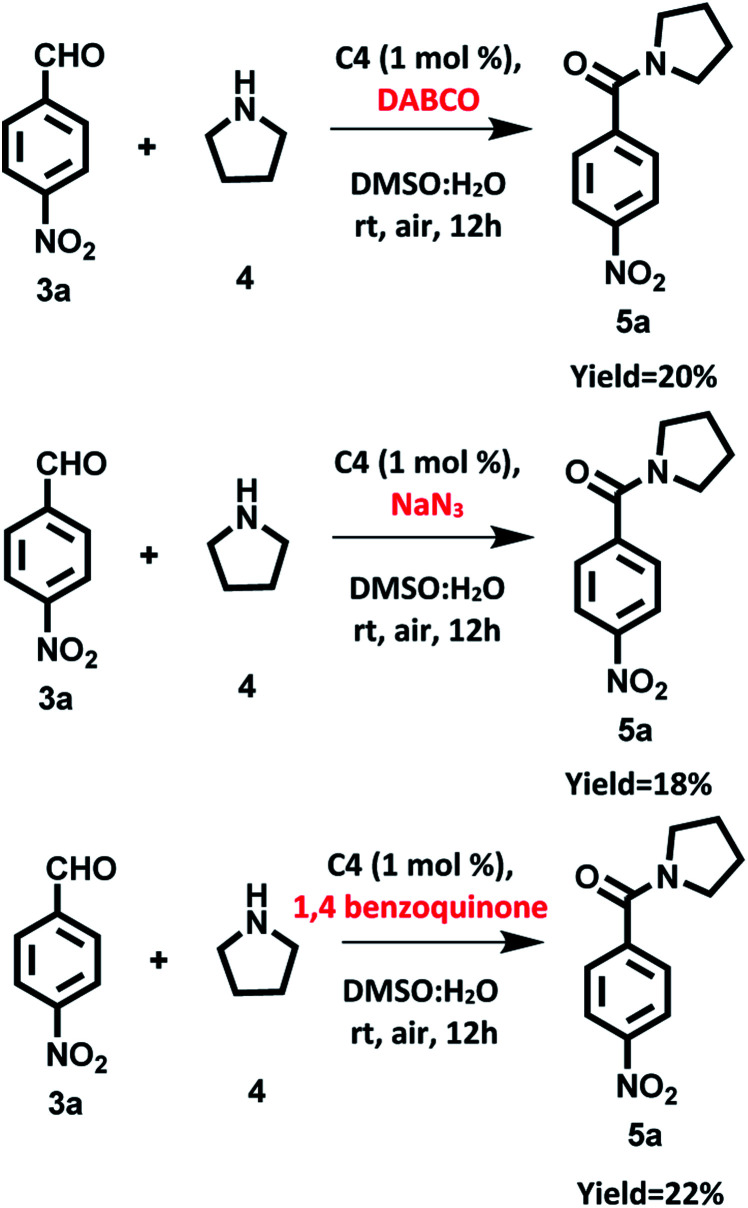
Oxidation amidation of 4-nitrobenzaldehyde with pyrrolidine in the presence of ROS quenchers using photocatalyst based on hemicyanine derivative (C4) under irradiation of visible light at room temperature and aerial conditions.

To confirm our assumption regarding the involvement of singlet oxygen/superoxide in reaction, we carried out the model reaction in the presence of singlet oxygen scavenger such as DABCO (1,4-diazabicyclo[2.2.2]-octane), sodium azide and in presence of superoxide scavenger such as 1,4-benzoquinone. We found that the photocatalytic reaction was affected significantly and the desired product was obtained in 20%, 18% and 22% yields respectively (Scheme 9). These observations support that both ^1^O_2_ and O_2_˙^−^ are involved in the photocatalytic reaction.

## Conclusion

Hemicyanine derivatives C1–C4 have been synthesized and utilized as photocatalysts in oxidative amidation of aromatic aldehydes under mild conditions. Among all the synthesized derivatives, C4 exhibited high efficiency which is attributed to its strong absorption in visible region, good water solubility, high singlet oxygen quantum yield (*Φ*_Δ_ = 0.85), efficient intersystem crossing and high ability to transport electron from donor to acceptor. All the mechanism investigations show participation of reactive oxygen species generated by triplet organic photosensitizer in oxidative amidation of aldehyde. The mild reaction conditions such as aerial conditions, aqueous media, low catalytic loading and visible light irradiation with broad substrate scope make this approach practically useful and eco-friendly for the synthesis of amide bond. Findings of this work will inspire the designing of new of “metal free” photosensitizers as photocatalyst in organic transformations.

## Experimental section

### General experimental methods and materials^18^

The general experimental methods, quantum yield calculations, and materials used are the same as those reported earlier by us.

#### UV-vis and fluorescence experiment^18^

The stock solutions (10^−3^ M) of hemicyanine derivatives were prepared by dissolving 3.33 mg, 3.02 mg, 2.78 mg and 3.86 mg of compound C1, C2, C3 and C4 respectively in 10.0 mL of DMSO. 15.0 μL of this stock solution further diluted with add 1485 μL of DMSO and 1500 μL of water (pH = 7.05) prepare 3.0 mL solution of derivative C1–C4 (5.0 μM) and this solution was used for each UV-vis and fluorescence experiments.

#### Determination of singlet oxygen quantum yield

Singlet oxygen quantum yields of derivatives C1 and C4 were obtained by the measurement of quenching of DPBF in the presence of C1 and C4 as photosensitizers taking the reference of methylene blue under same conditions (*Φ*_Δ_ = 0.52) in DMSO as solvent. The irradiation was done in a fluorescence spectrophotometer equipped with a Xe lamp at the excitation wavelength of C1 and C2 for 50 min. UV-visible measurements were performed using UV-vis spectrophotometer. All the experiments were carried out at 25 °C.

#### Synthesis of derivative C2

To a the mixture of 1,2,3,3-tetramethyl-3*H*-indol-1-ium (205 mg, 0.68 mmol) and 2, 7-azaindole-3-carboxaldehyde (100 mg, 0.68 mmol) in anhydrous ethanol (20.0 mL) were added two drops of piperidine. Then the reaction mixture was refluxed for 48 h under inert atmosphere. After completion of reaction, red coloured solid was filtered and washed with ethanol (yield = 80%). ^1^H NMR (300 MHz, DMSO-*d*_6_) *δ* = 13.29 (s, 1H, NH), 8.87–8.83 (m, 2H), 8.74 (d, *J* = 15 Hz, 2H), 8.54 (d, *J* = 6 Hz, 2H), 7.90 (t, *J* = 7.5 Hz, 2H), 7.69–7.59 (m, 2H), 7.52–7.48 (m, 1H), 7.31 (d, *J* = 15 Hz, 2H), 4.13 (s, 3H), 1.89 (s, 6H). ^13^C NMR (125 MHz, DMSO-*d*_6_) *δ* = 181.15, 151.00, 148.90, 145.71, 143.14, 140.44, 130.52, 129.45, 123.16, 119.11, 114.41, 106.93, 56.50; mass, *m*/*z* = 302.16 [M + 1]^+^.

### General procedure for oxidative amidation of aromatic aldehyde using hemicyanine derivative under visible light irradiation

The mixture of aromatic aldehydes (0.2 mmol, 1.0 equiv.) and amine (0.6 mmol, 3.0 equiv.) in DMSO : H_2_O (1 : 1) in presence of C4 (1 mol%) as a photocatalyst was stirred at room temperature for 12 h under aerial condition and visible light irradiation (for products 5a, 6a, 8a, 7e, 5e), 20 h (for 5c, 5d, 7c, 7d) and 30 h (for substrate 5b, 7b, 8b). After the completion of the reaction (monitored by TLC), organic part was extracted with EtOAc. The organic layer dried over anhydrous sodium sulphate and concentrate under reduced pressure. The crude was purified by column chromatography using EtOAc : hexane (1 : 1) to furnished the desired products. All the products were identified by ^1^H NMR spectroscopy (Fig. S25–S36 in the ESI†).

### Preparation of photocatalyst coated dip strip

The filter paper strip was dipped in DMSO solution of C4 photocatalyst (10.0 μM) for 1 h. Afterwards, removed the filter paper and air dried. The dried filter paper strip has been used for carrying out the oxidative amidation of the aromatic aldehydes under visible light.

## Conflicts of interest

There are no conflicts to declare.

## Supplementary Material

RA-008-C8RA06232C-s001
