# Supplementary material for: Exploring organic photosensitizers based on hemicyanine derivatives: a sustainable approach for preparation of amide linkages
Source: RSC Adv. 2018 Sep 5;8(54):31237–45. doi: 10.1039/c8ra06232c (PMC9085617; doi:10.1039/c8ra06232c)
Supplement: RA-008-C8RA06232C-s001 [file RA-008-C8RA06232C-s001.pdf]

Electronic Supplementary Material (ESI) for RSC Advances.
This journal is © The Royal Society of Chemistry 2018

Supplementary Information

Exploring Organic Photosensitizers Based on Hemicyanine Derivatives: A Sustainable Approach for Preparation of Amide Linkages

*Harnimarta Deol, Manoj Kumar, Vandana Bhalla**

Department of Chemistry, UGC Sponsored Centre for Advanced Studies-II

Guru Nanak Dev University, Amritsar 143005, and Punjab, India

vanmanan@yahoo.co.in.

Table of Contents

S3: Comparison table of **C4** photocatalyst with off shelf photosensitizers for carrying out oxidative amidation of aldehydes.

S4: Uv-vis spectra of **C1, C2, C3** and **C4** in DMSO: H_2O (1:1) solvent mixture.

S4: Fluorescence spectra of **C1, C2, C3** and **C4** in DMSO:H_2O (1:1) solvent mixture.

S5: Fluorescence lifetime decay profile of C1.

S5: Fluorescence lifetime decay profile of C2.

S6: Fluorescence lifetime decay profile of C3.

S6: Fluorescence lifetime decay profile of C4.

S7: (a) Uv-vis spectra of DPBF in presence of C1. (b) Semilogarithmic plots for the absorption decays of DPBF (50μM) (lnA_0/A) versus time .

S7: (a) Uv-vis spectra of DPBF in presence of C4. (b) Semilogarithmic plots for the absorption decays of DPBF (50μM) (lnA0/A) versus time.

S8: (A) UV-vis and (B) fluorescence spectra of derivative **C1** after irradiation of 36 h.

S8: (A) UV-vis and (B) fluorescence spectra of derivative **C2** after irradiation of 36 h.

S9: (A) UV-vis and (B) fluorescence spectra of derivative **C3** after irradiation of 36 h

S9: (A) UV-vis and (B) fluorescence spectra of derivative **C4** after irradiation of 36 h.

S10: Uv-vis spectra of methyl viologen in presence of C1.

S10: Uv-vis spectra of methyl viologen in presence of C2.

S11: Uv-vis spectra of methyl viologen in presence of C3.

S11: Fluorescence spectra of derivative **C4** upon addition of pyrrolidine in DMSO:Water.

S12: Fluorescence spectra of derivative **C1** upon addition of pyrrolidine in DMSO:Water.

S12: Fluorescence spectra of derivative **C2** upon addition of pyrrolidine in DMSO:Water.

S13: Fluorescence spectra of derivative **C3** upon addition of pyrrolidine in DMSO:Water.

S13: Oxidative amidation of nitrobenzaldehyde in presence of solar light using **C4** as photosensitizer under optimized conditions.

S14: Images showing the recovery of photocatalyst **C4** by usual work up.

S14: Reusability of **C4** for carrying out oxidative amidation of aromatic aldehydes under visible light irradiation..

S15: Whatman filter paper (b) coated with derivative C4 by dip the whatman filter paper into solution of derivative **C4.**

S15: Test for detection of generation of H_2O_2.

S18: ^{1}H NMR spectrum of **5a**.

S19: ^{1}H NMR spectrum of **5b**.

S20: ^{1}H NMR spectrum of **5c**.

S21: ^{1}H NMR spectrum of **5d**.

S22: ^{1}H NMR spectrum of **5e**.

S23: ^{1}H NMR spectrum of **7a**.

S24: ^{1}H NMR spectrum of **7b**.

S25: ^{1}H NMR spectrum of **7c**.

S26: ^{1}H NMR spectrum of **7d**.

S27: ^{1}H NMR spectrum of **7e**.

S28: ^{1}H NMR spectrum of **8a**.

S29: ^{1}H NMR spectrum of **8b**.

S30: ^{1}H NMR spectrum of **C2**.

S31: ^{13}C NMR spectrum of **C2**.

S32: Mass spectrum of **C2**.

S33: ^{1}H NMR spectrum of **C3**.

S34: ^{13}C NMR spectrum of **C3**.

S35: Mass spectrum of **C3**.

S36: ^{1}H NMR spectrum of **C4**.

S37: Mass spectrum of **C4**.

Table S1. Comparison of C4 photocatalyst with off shelf photosensitizer for carrying out oxidative amidation of aldehydes.

Photocatalysts	Solvent	Time	Additive	Reuseability/ Recyclability	Yield
**C4 (This Work)**	**DMSO:H_2O (1:1)**	**12h**	**-**	**Yes**	**82%**
Phenazine ethosulfate	MeCN	12h	-	No	41%
$Ru(bpy)_3Cl_2$	MeCN	12h	-	No	37%
$Ru(phen)_3Cl_2$	MeCN	12h	-	No	46%
$Ru(phen)_3(PF_6)_2$	MeCN	12h	-	No	42%
$Ir(dtbpy)(ppy)_2PF_6$	MeCN	12h	-	No	44%
Nile red	MeCN	12h	-	No	29%
Rhodamine B	MeCN	12h	-	No	26%
Alizarin red S	MeCN	12h	-	No	22%
Methylene blue	MeCN	12h	-	No	16%

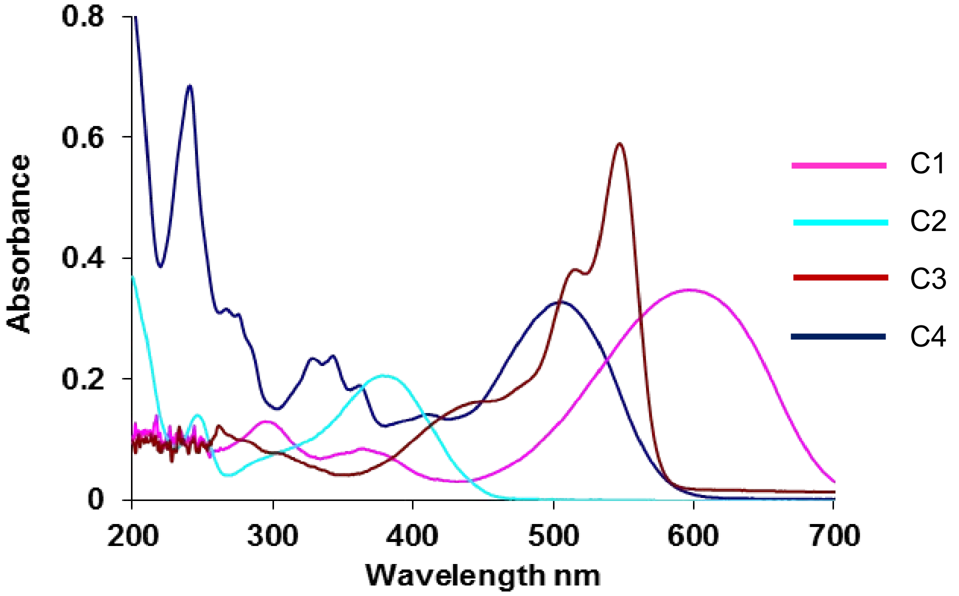

Fig. S1. Uv-vis spectra of **C1, C2, C3** and **C4** in DMSO: H_2O (1:1) solvent mixture.

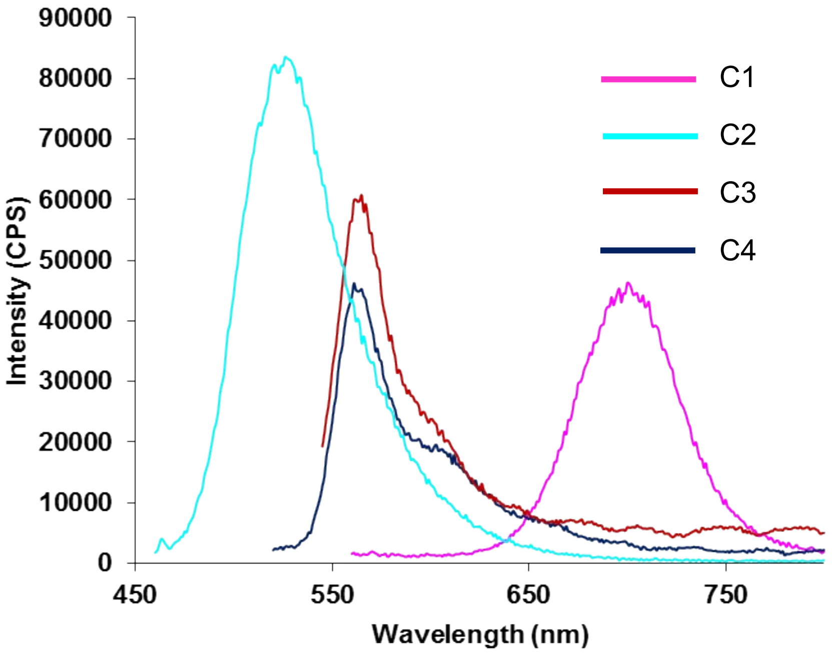

Fig. S2. Fluorescence spectra of **C1, C2, C3** and **C4** in DMSO:H_2O (1:1) solvent mixture.

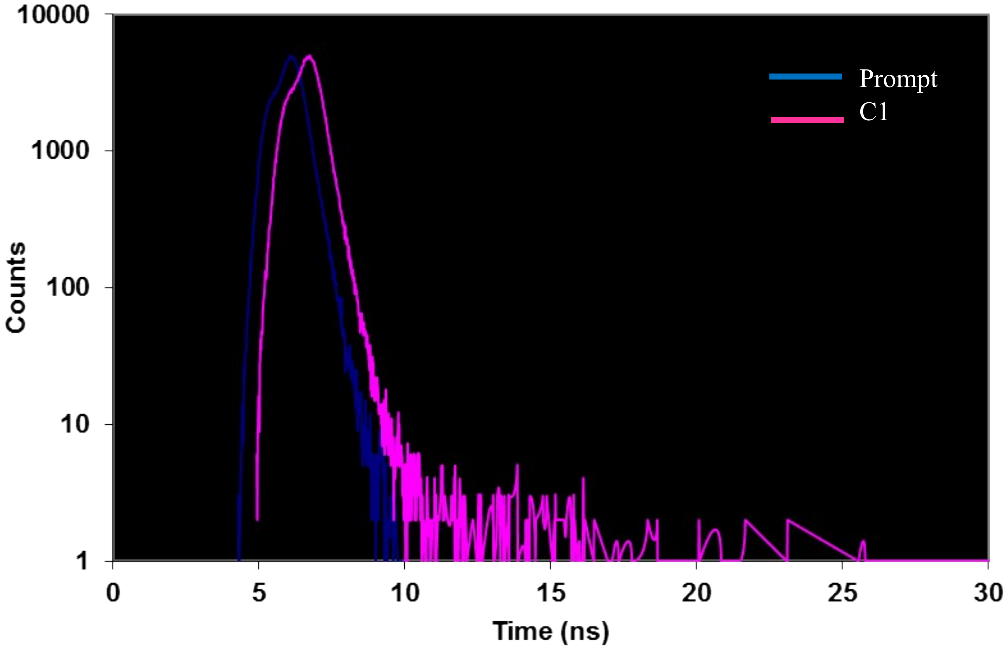

Fig. S3 Fluorescence lifetime decay profiles of **C1** in H_2O: DMSO (1:1, v/v). IRF = instrument response function. λ_{ex} = 635 nm and emission spectra are recorded at 686 nm with 32 slit width.

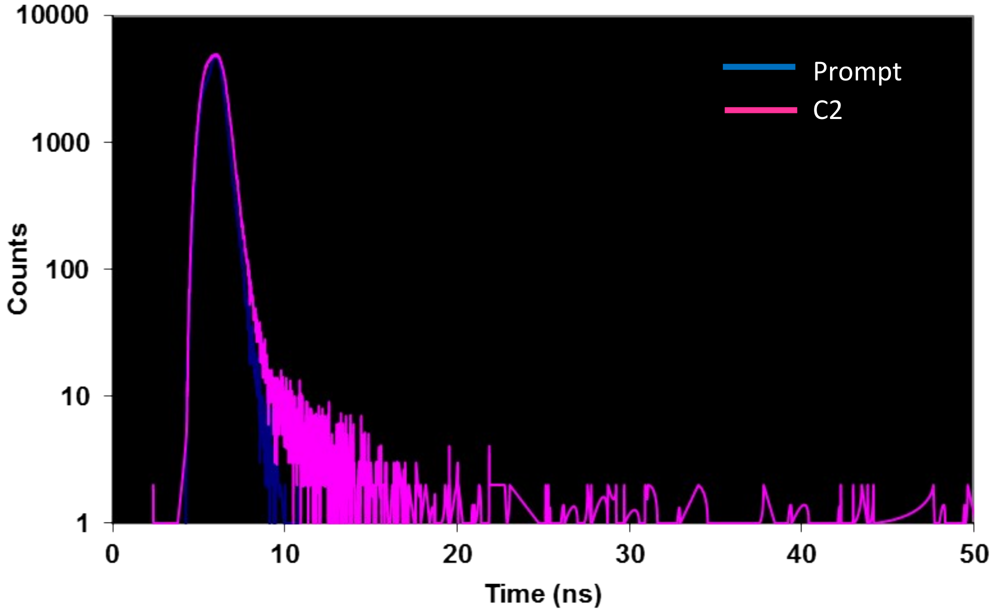

Fig. S4 Fluorescence lifetime decay profiles of **C2** in H_2O: DMSO (1:1, v/v). IRF = instrument response function. λ_{ex} = 485 nm and emission spectra are recorded at 511 nm with 32 slit width.

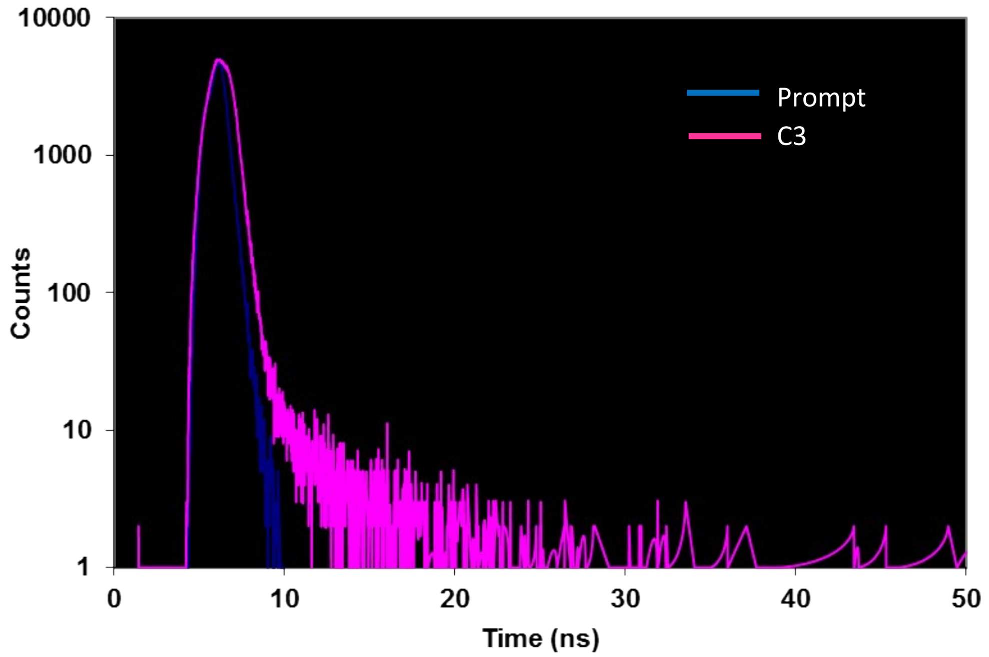

Fig. S5 Fluorescence lifetime decay profiles of **C3** in H_2O: DMSO (1:1, v/v). IRF = instrument response function. λ_{ex} = 485 nm and emission spectra are recorded at 557 nm with 32 slit width.

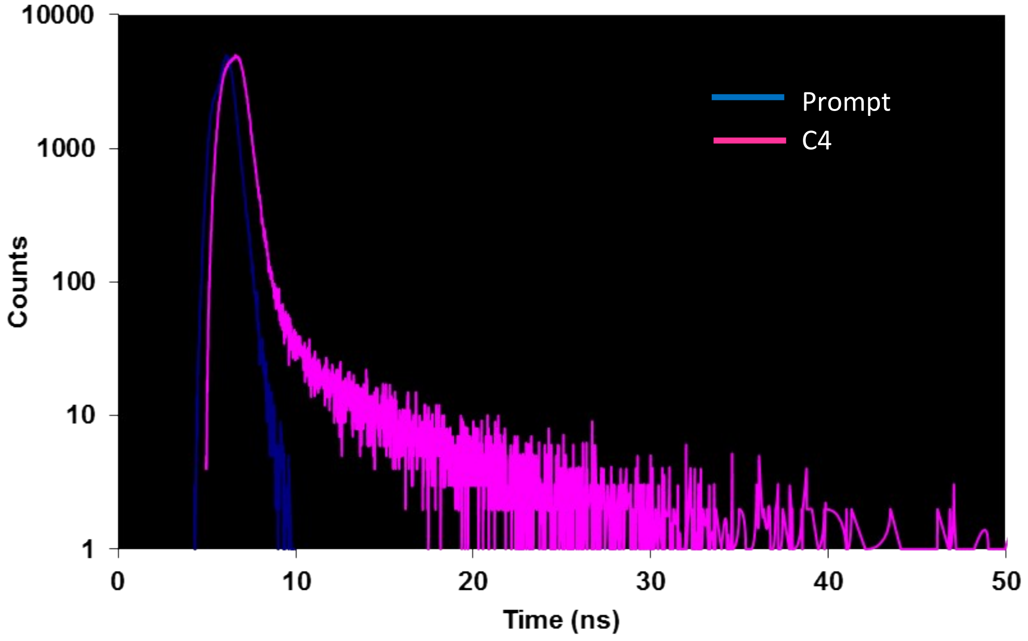

Fig. S6 Fluorescence lifetime decay profiles of **C4** in H_2O: DMSO (1:1, v/v). IRF = instrument response function. λ_{ex} = 485 nm and emission spectra are recorded at 616 nm with 32 slit width.

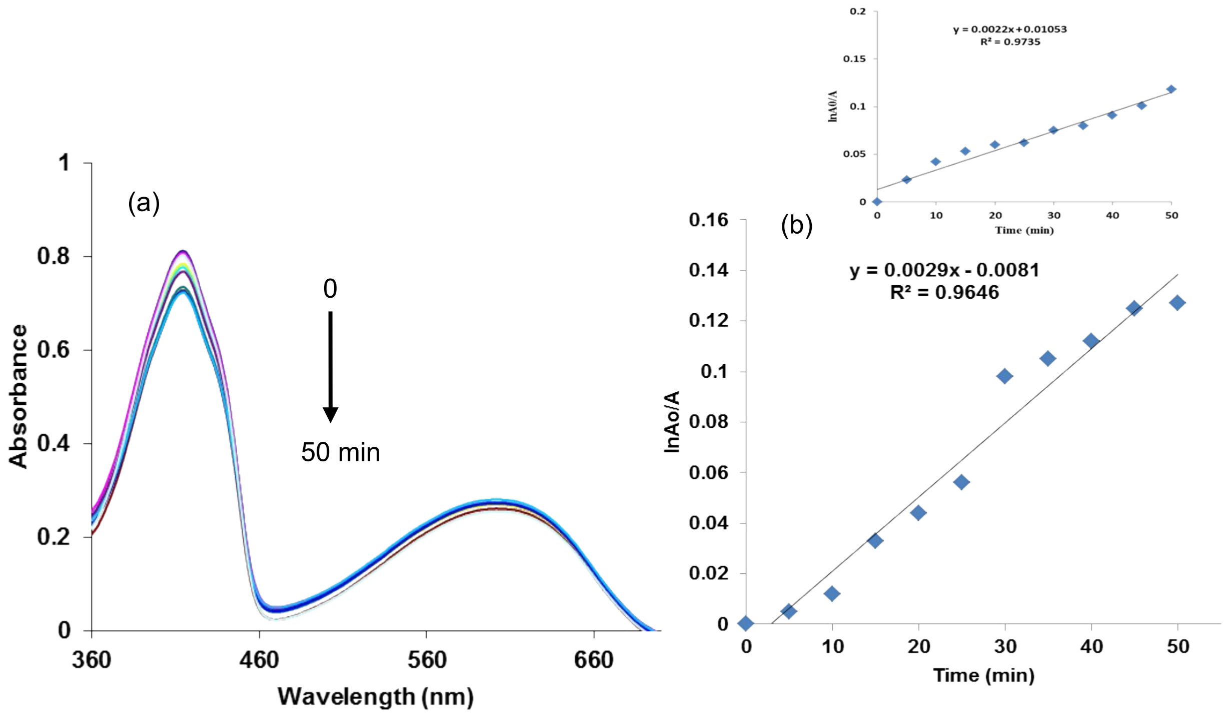

Fig. S7 (a) Decrease in absorption maxima of DPBF (50μM) at 418 nm in DMSO under illumination in the presence of 5μM **C1** for 50 min after the solution was saturated with air oxygen. (b) Semilogarithmic plots for the absorption decays of DPBF (50μM) (lnA_0/A) versus time at the same experimental conditions. inset: shows semilogarithmic plots for reference dye methylene blue under same conditions.

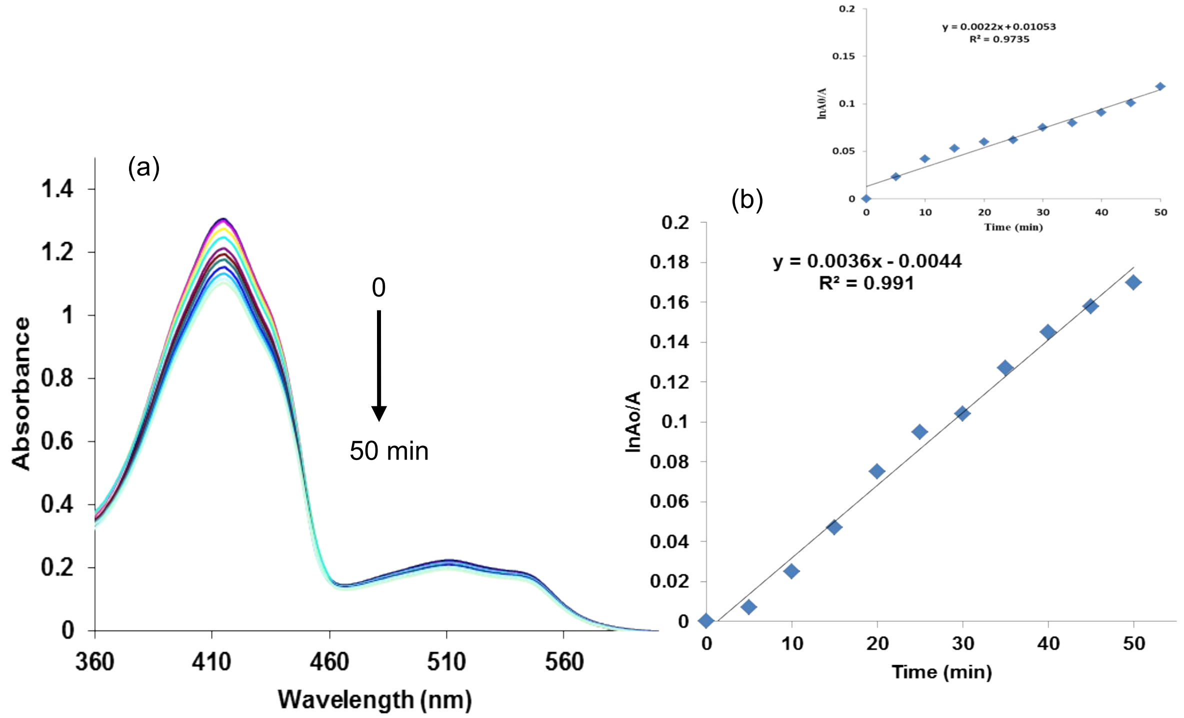

Fig. S8 (a) Decrease in absorption maxima of DPBF (50μM) at 418 nm in DMSO under illumination in the presence of 5μM **C4** for 50 min after the solution was saturated with an air oxygen. (b) Semilogarithmic plots for the absorption decays of DPBF (50μM) (lnA0/A) versus time at the same experimental conditions. inset: shows semilogarithmic plots for reference dye methylene blue under same conditions.

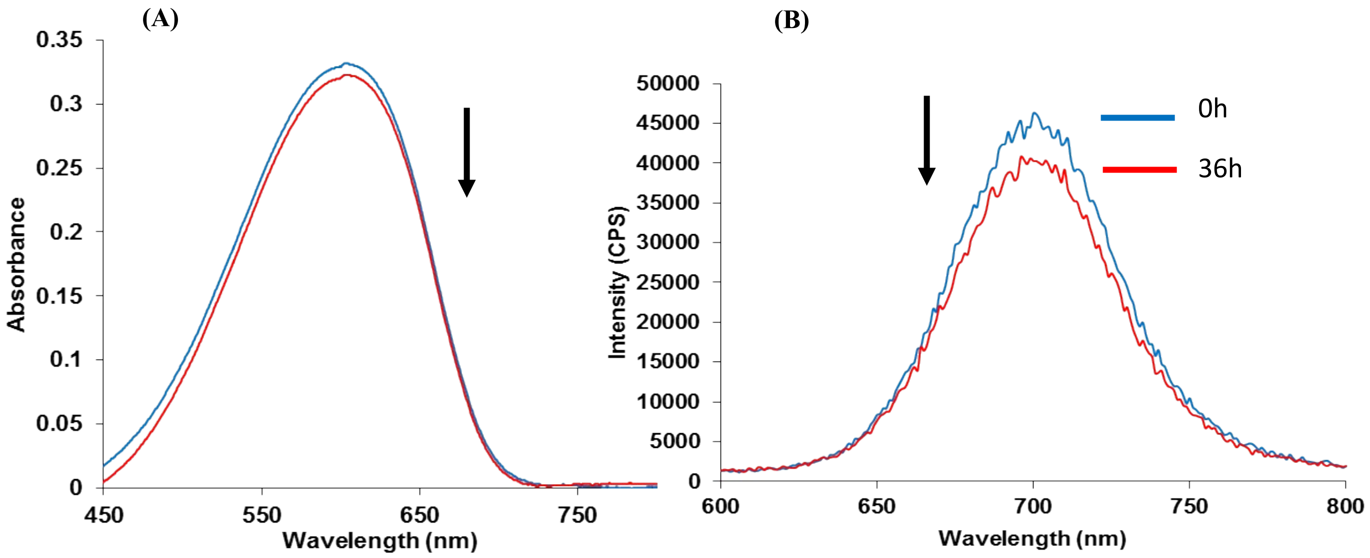

Fig. S9 (A) UV-vis and (B) fluorescence spectra of derivative **C1** after irradiation of 36h.

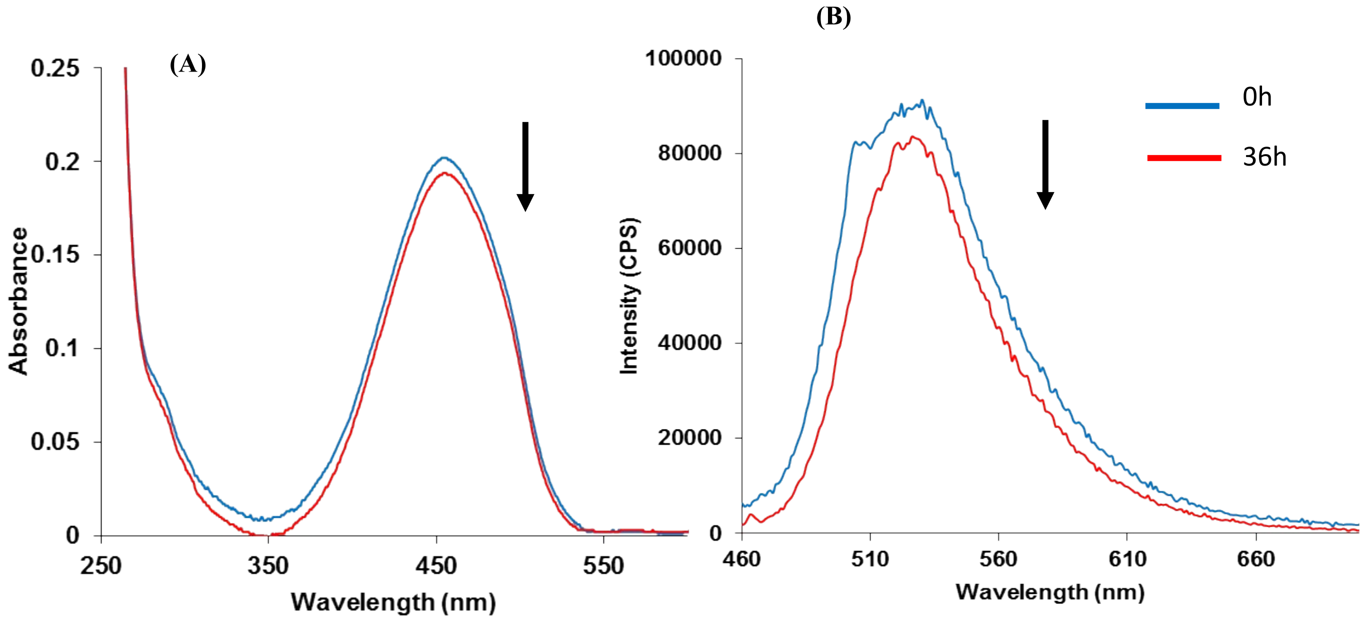

Fig. S10 (A) UV-vis and (B) fluorescence spectra of derivative **C2** after irradiation of 36h.

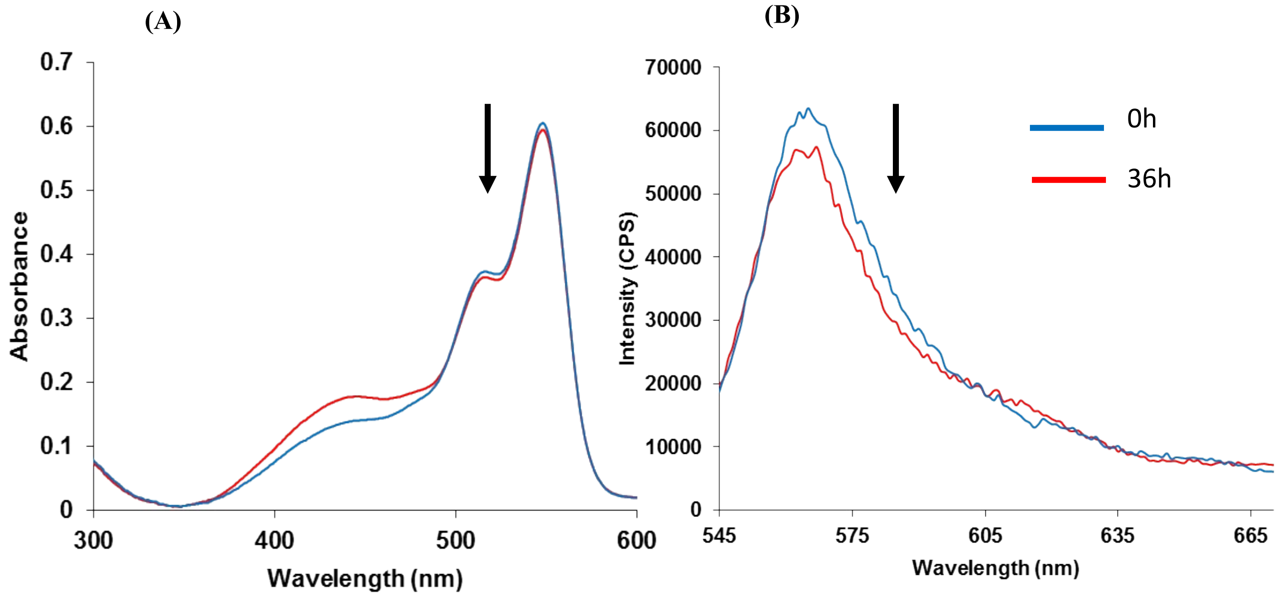

Fig. S11 (A) UV-vis and (B) fluorescence spectra of derivative **C3** after irradiation of 36h.

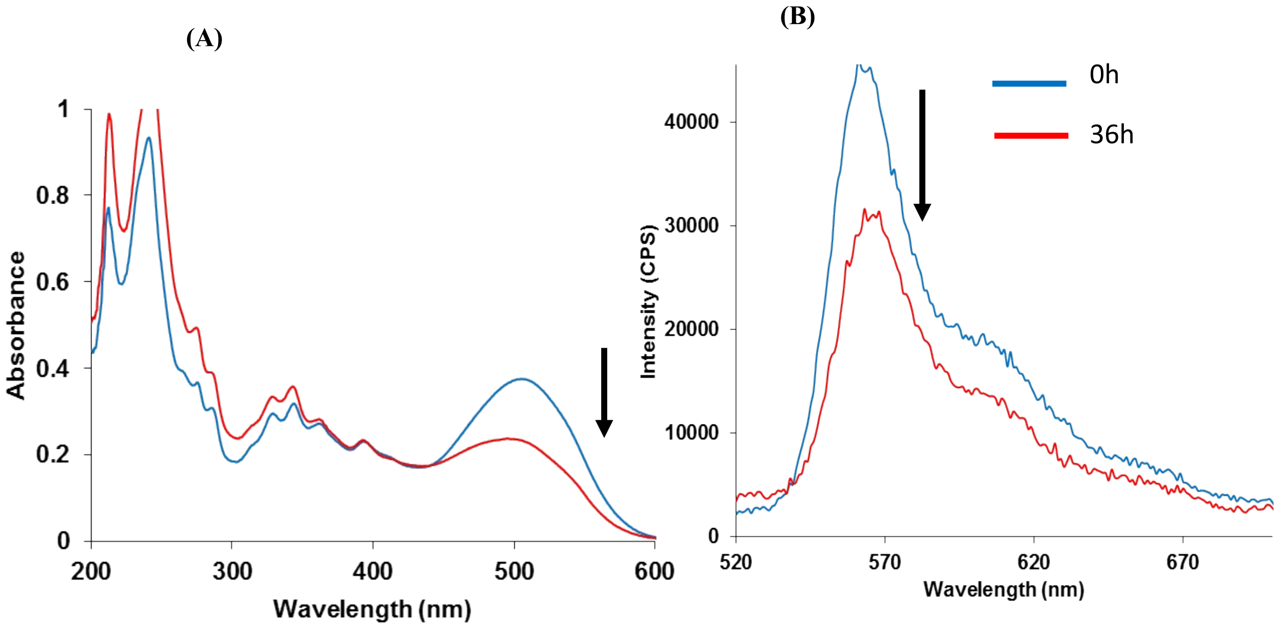

Fig. S12 (A) Uv-vis and (B) fluorescence spectra of derivative **C1** after irradiation of 36h.

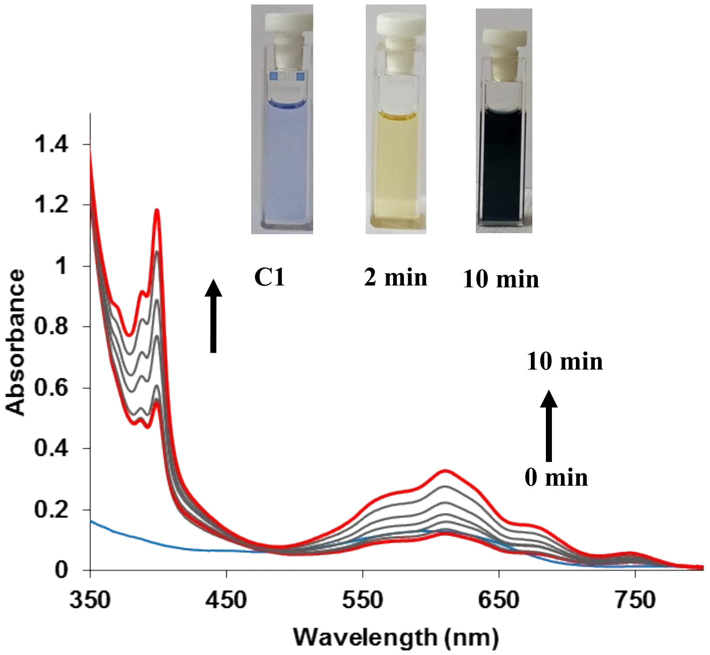

Fig. S13 Absorption spectral changes of derivative **C1** (0.02 mM) in presence of MV^{2+} (0.2 mM) and TEOA (50 mM) in DMSO under room light and inert atmosphere. inset: Photograph of solution before and after 10 min.

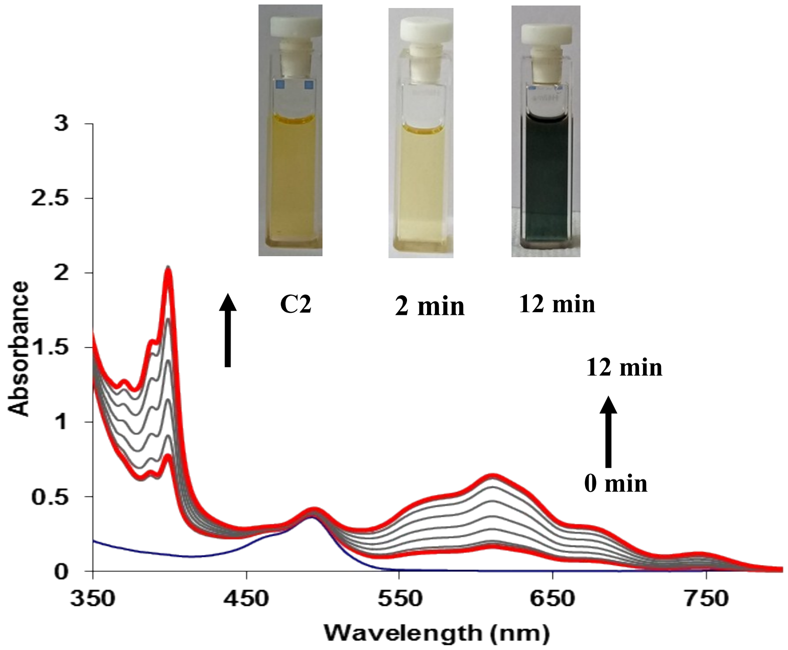

Fig. S14 Absorption spectral changes of derivative **C2** (0.02 mM) in presence of MV^{2+} (0.2 mM) and TEOA (50 mM) in DMSO under room light and inert atmosphere. inset: Photograph of solution before and after 12 min.

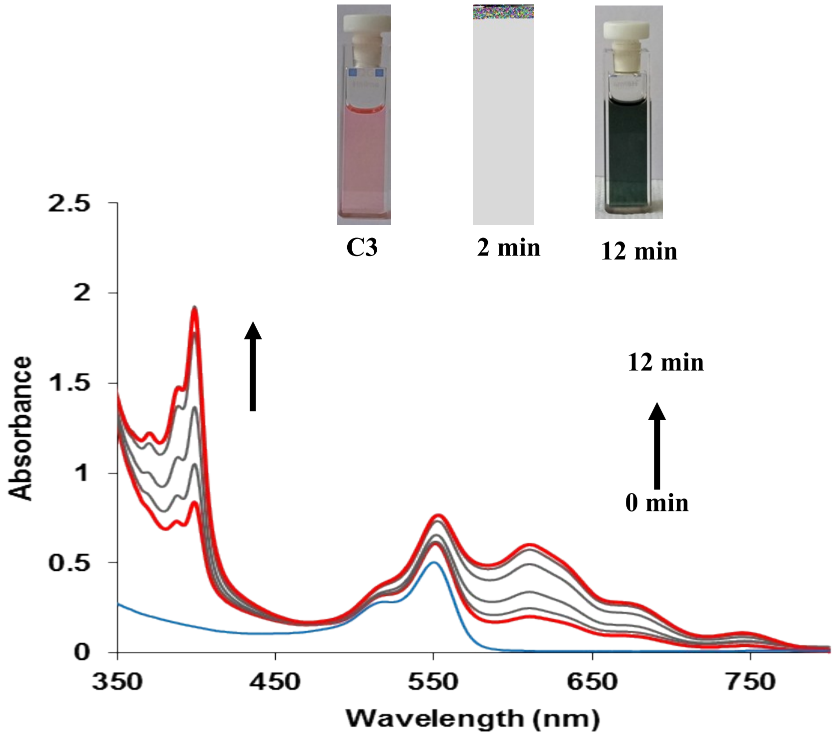

Fig. S15 Absorption spectral changes of derivative **C3** (0.02 mM) in presence of MV^{2+} (0.2 mM) and TEOA (50 mM) in DMSO under room light and inert atmosphere. inset: Photograph of solution before and after 12 min.

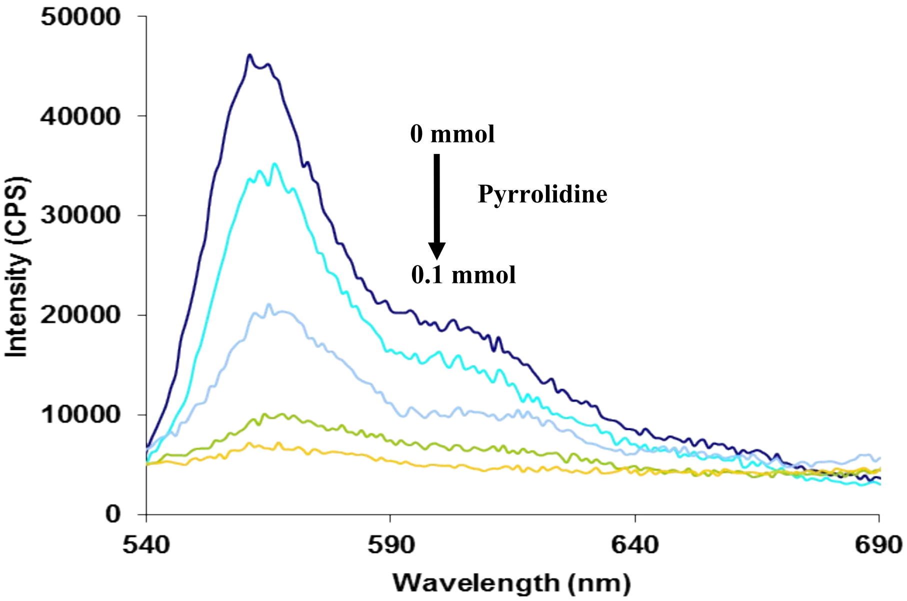

Fig. S16 Fluorescence spectra of derivative **C4** (5μM) upon addition of pyrrolidine (0.1 mmol) in DMSO:Water (1:1).

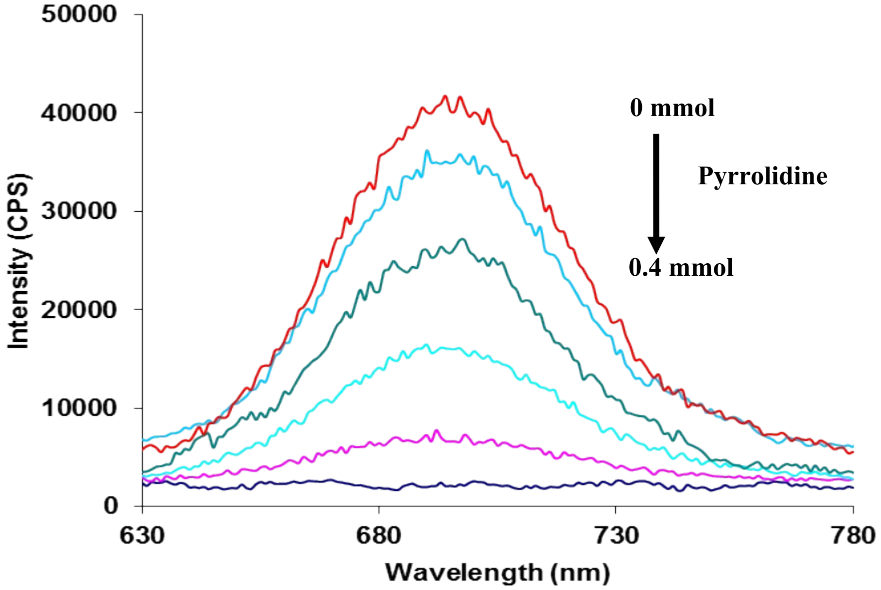

Fig. S17 Fluorescence spectra of derivative **C1** (5µM) upon addition of pyrrolidine (0.4 mmol) in DMSO:Water (1:1).

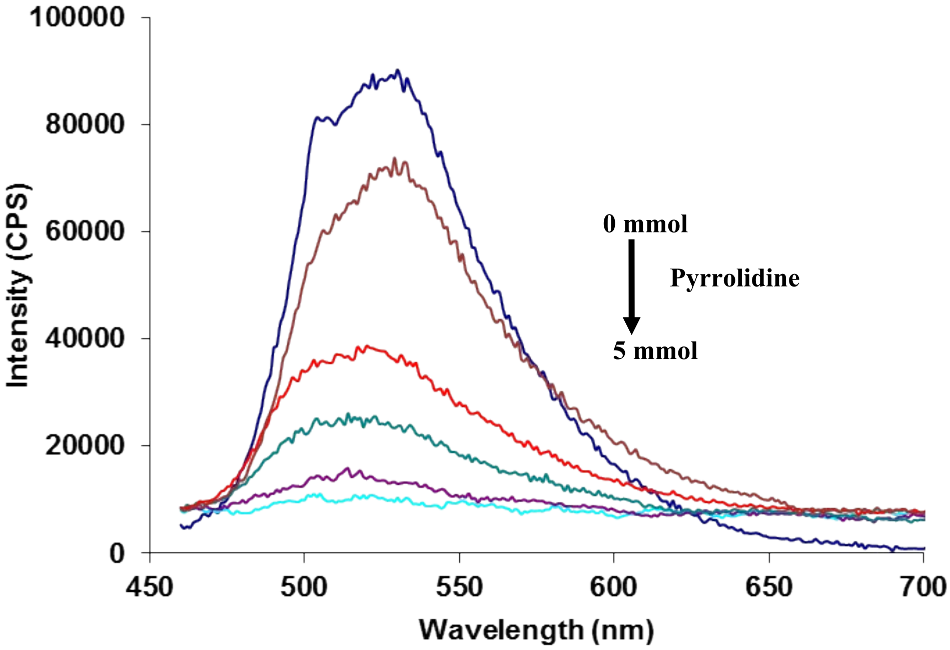

Fig. S18 Fluorescence spectra of derivative **C2** (5µM) upon addition of pyrrolidine (5 mmol) in DMSO:Water (1:1).

`

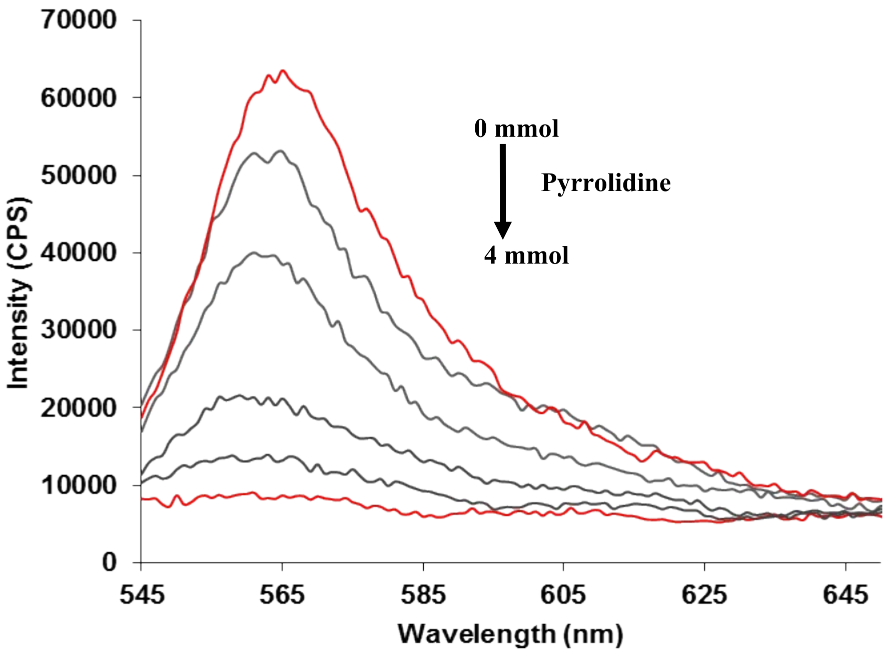

Fig. S19 Fluorescence spectra of derivative **C3** (5μM) upon addition of pyrrolidine (4 mmol) in DMSO:Water (1:1).

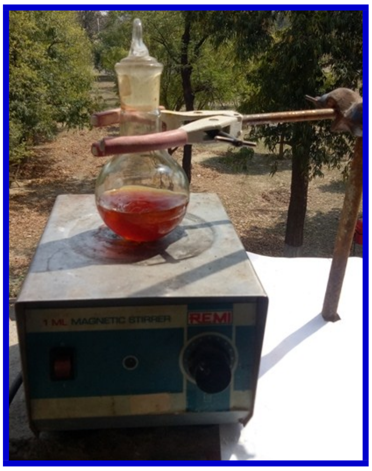

Fig. S20 Oxidative amidation of nitrobenzaldehyde in presence of solar light using **C4** as photosensitizer under optimized conditions.

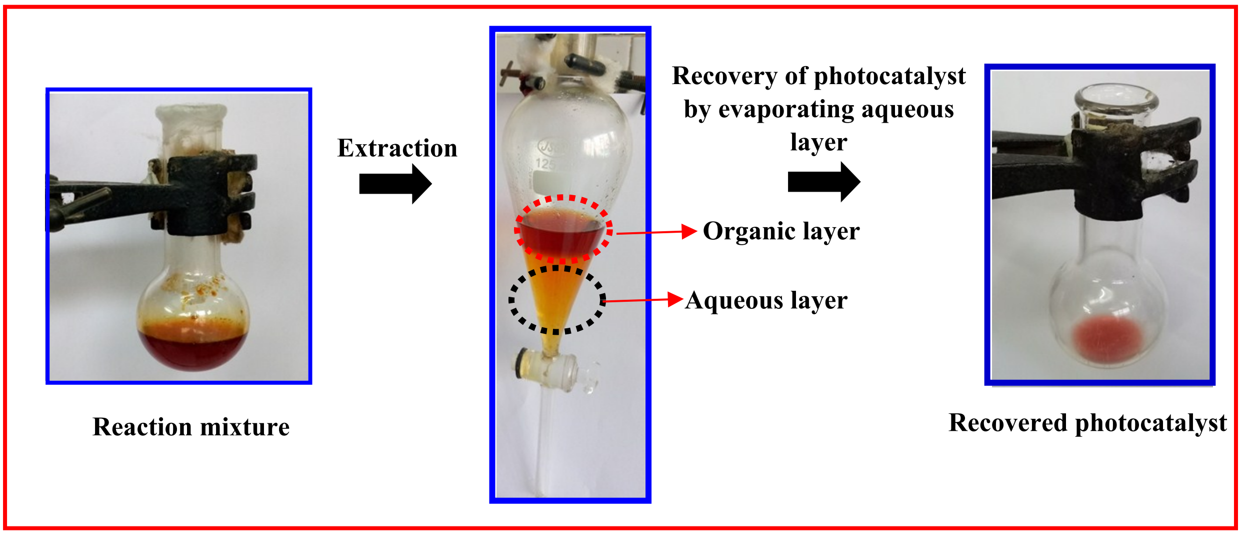

Fig. S21 Images showing the recovery of photocatalyst **C4** by usual work up.

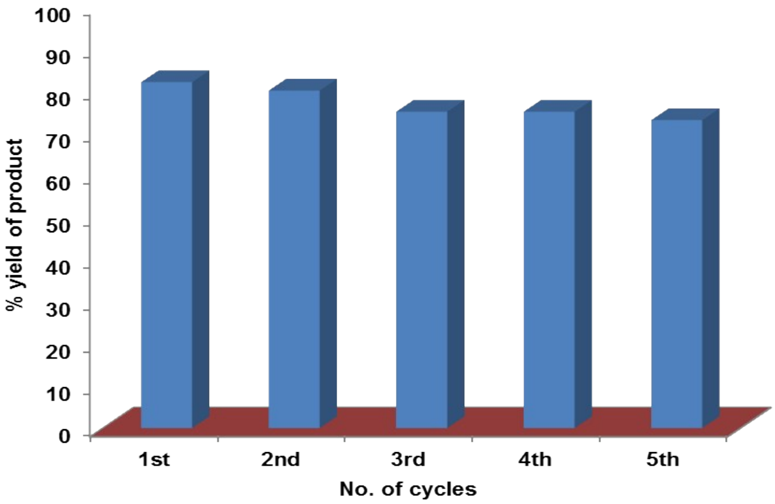

Fig. S22 Reusability of **C4** for carrying out oxidative amidation of aromatic aldehydes under visible light irradiation.

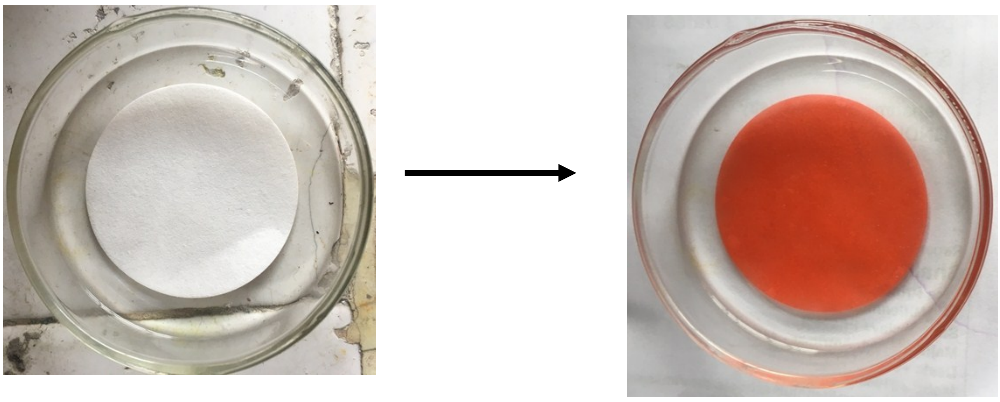

Fig. S23 (a) Whatman filter paper (b) coated with derivative C4 by dip the whatman filter paper into solution of derivative **C4** (5 μM).

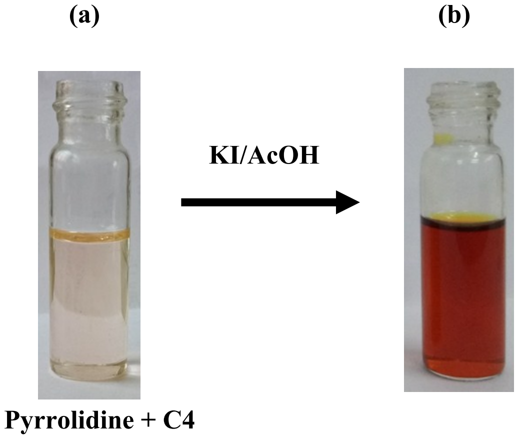

Fig. S24 (a) Reaction mixture of pyrrolidine (0.75 mmol) and **C4** (1 mol%) after irradiation of 8 h**;** (b) After addition of mixture KI (1.0×10^{-1} M), aqueous acetic acid (1.0×10^{-1} M) colour of solution changes to brown.

(4-nitrophenyl)(pyrrolidin-1-yl)methanone[1], 5a

^{1}H NMR (300 MHz, $CDCl_3$): δ (ppm) = 8.27 (d, J = 9 Hz, 2H, Ar-H), 7.68 (d, J = 9 Hz, 2H, Ar-H), 3.66 (t, J = 6 Hz, 2H, CH_2), 3.38 (t, J = 6 Hz, 2H, CH_2), 2.01-1.90 (m, 4H, CH_2).

(4-methoxyphenyl)(pyrrolidin-1-yl)methanone[2], 5b

^{1}H NMR (300 MHz, $CDCl_3$): δ (ppm) = 7.51 (d, J = 6 Hz, 2H, Ar-H), 6.92 (d, J = 6 Hz, 2H, Ar-H), 3.83 (s, 3H, OCH_3), 3.63 (t, J = 6 Hz, 2H, CH_2), 3.48 (t, J = 9 Hz, 2H, CH_2),1.94-1.86 (m, 4H, CH_2).

Phenyl(pyrrolidin-1-yl)methanone[1], 5c

^{1}H NMR (500 MHz, $CDCl_3$): δ (ppm) = 7.51-7.50 (m, 2H, Ar-H), 7.40-7.38 (m, 3H, Ar-H), 3.64 (t, J = 7.5 Hz, 2H, CH_2), 3.42 (t, J = 7.5 Hz, 2H, CH_2), 1.98-1.93 (m, 2H, CH_2), 1.89-1.84 (m, 2H, CH_2).

(4-cyanophenyl)(pyrrolidin-1-yl)methanone[1], 5d

^{1}H NMR (500 MHz, $CDCl_3$): δ (ppm) = 7.68 (d, J = 10 Hz, 2H, Ar-H), 7.59 (d, J = 10 Hz, 2H, Ar-H), 3.62 (t, J = 7.5 Hz, 2H, CH_2), 3.35 (t, J = 7.5 Hz, 2H, CH_2), 1.98-1.93 (m, 2H, CH_2), 1.91-1.86 (m, 2H, CH_2).

(4-chlorophenyl)(pyrrolidin-1-yl)methanone[1], 5e

^{1}H NMR (300 MHz, $CDCl_3$): δ (ppm) = 7.43 (d, J = 6 Hz, 2H, Ar-H), 7.33 (d, J = 6 Hz, 2H, Ar-H), 3.59 (t, J = 6 Hz, 2H, CH_2), 3.37 (t, J = 6Hz, 2H, CH_2), 1.94-1.82 (m, 4H, CH_2).

(4-nitrophenyl)(piperidin-1-yl)methanone[3], 7a

^{1}H NMR (400 MHz, $CDCl_3$): δ (ppm) = 8.27 (d, J = 8 Hz, 2H, Ar-H), 7.55 (d, J = 8 Hz, 2H, Ar-H), 3.73 (bs, 2H, CH_2), 3.28 (bs, 2H, CH_2), 1.79-1.52 (m, 6H, CH_2).

(4-methoxyphenyl)(piperidin-1-yl)methanone[2], 7b

^{1}H NMR (300 MHz, $CDCl_3$): δ (ppm) = 7.37 (d, J = 9 Hz, 2H, Ar-H), 6.90 (d, J = 9 Hz, 2H, Ar-H), 3.83 (s, 3H, OCH_3), 3.64 (br, 2H, CH_2), 3.44 (br, 2H, CH_2), 1.66-1.58 (m, 6H, CH_2).

Phenyl (piperidin-1-yl)methanone, 7c

^{1}H NMR (300 MHz, $CDCl_3$): δ (ppm) = 7.37 (m, 5H, Ar-H), 3.69 (br, 2H, CH_2), 3.33 (br, 2H, CH_2), 1.66-1.51 (m, 6H, CH_2).

(4-cyanophenyl)(piperidin-1-yl)methanone[1], 7d

^{1}H NMR (300 MHz, $CDCl_3$): δ (ppm) = 7.70 (d, J = 6 Hz, 2H, Ar-H), 7.48 (d, J = 6 Hz, 2H, Ar-H), 3.71 (br, 2H, CH_2), 3.27 (br, 2H, CH_2), 1.60 (bs, 4H, CH_2), 1.53 (br, 2H, CH_2).

(4-chlorophenyl)(piperidin-1-yl)methanone[2], 7e

^{1}H NMR (500 MHz, $CDCl_3$): δ (ppm) = 7.38-7.32 (m, 4H, Ar-H), 3.69 (bs, 2H, CH_2), 3.33 (bs, 2H, CH_2), 1.68-1.51 (m, 6H, CH_2).

morpholino(4-nitrophenyl)methanone[2], 8a

^{1}H NMR (400 MHz, $CDCl_3$): δ (ppm) = 8.27 (d, J = 8 Hz, 2H, Ar-H), 7.57 (d, J = 8 Hz, 2H, Ar-H), 3.79-3.37 (m, 8H, CH_2).

morpholino(4-methoxyphenyl)methanone[2], 8b

^{1}H NMR (300 MHz, $CDCl_3$): δ (ppm) = 7.28 (d, J = 9 Hz, 2H, Ar-H), 6.82 (d, J = 9 Hz, 2H, Ar-H), 3.72 (s, 3H, OCH_3), 3.60 (br, 8H, CH_2).

Derivative C3.

^{1}H NMR (DMSO-d_6, 400 MHz,): δ (ppm) = 10.02 (s, 1H), 8.35 (d, J = 6 Hz, 1H), 8.12 (d, J = 8 Hz, 2H), 7.83 (d, J = 8 Hz, 2H), 7.62-7.50 (m, 2H), 7.45 (d, J = 16 Hz, 2H), 6.95 (d, J = 8 Hz, 2H), 4.07 (s, 3H), 1.76 (s, 6H), ^{13}C NMR (DMSO-d_6, 125 MHz) δ = 163.69, 154.24, 143.65, 142.33, 134.02, 129.32, 126.46, 123.23, 116.99, 115.11, 109.76, 52.20, 34.97, 26.14; MS (ESI): m/z 278.16 $[M]^+$.

Derivative C4.

^{1}H NMR (DMSO-d_6, 400 MHz): δ (ppm) = 9.27 (d, J = 16 Hz, 1H), 8.99 (d, J = 8 Hz, 1H), 8.74 (d, J = 8 Hz, 1H), 8.49-8.38 (m, 5H), 8.30 (d, J = 8 Hz, 1H), 8.19-8.14 (m, 1H), 7.98-7.90 (m, 3H), 7.65-7.63 (m, 2H), 4.24 (s, 3H), 1.91 (s, 6H), MS (ESI): m/z 386.48 $[M+1]^+$.

Refrences

1. X.-F. Wang, S.-S. Yu, C. Wang, D. Xue and J. Xiao, *Org. Biomol. Chem.*, 2016, **14**, 7028.

2. K. Ekoue-Kovi and C. Wolf, *Org. Lett.*, 2007, **9**, 3429.

3. Y.-C. Hsu, V. C.-C. Wang, K.-C. A. Yeung, C.-Y. Tsai, C.-C. Chang, B.-C. Lin, Y.-T. Chan, C.-P. Hsu, G. P. A. Yap, T. Jurca and T.-G. Ong, *Angew. Chem. Int. Ed.*, 2018, **57**, 4622.

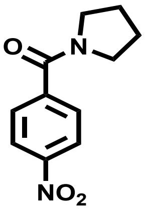

5a

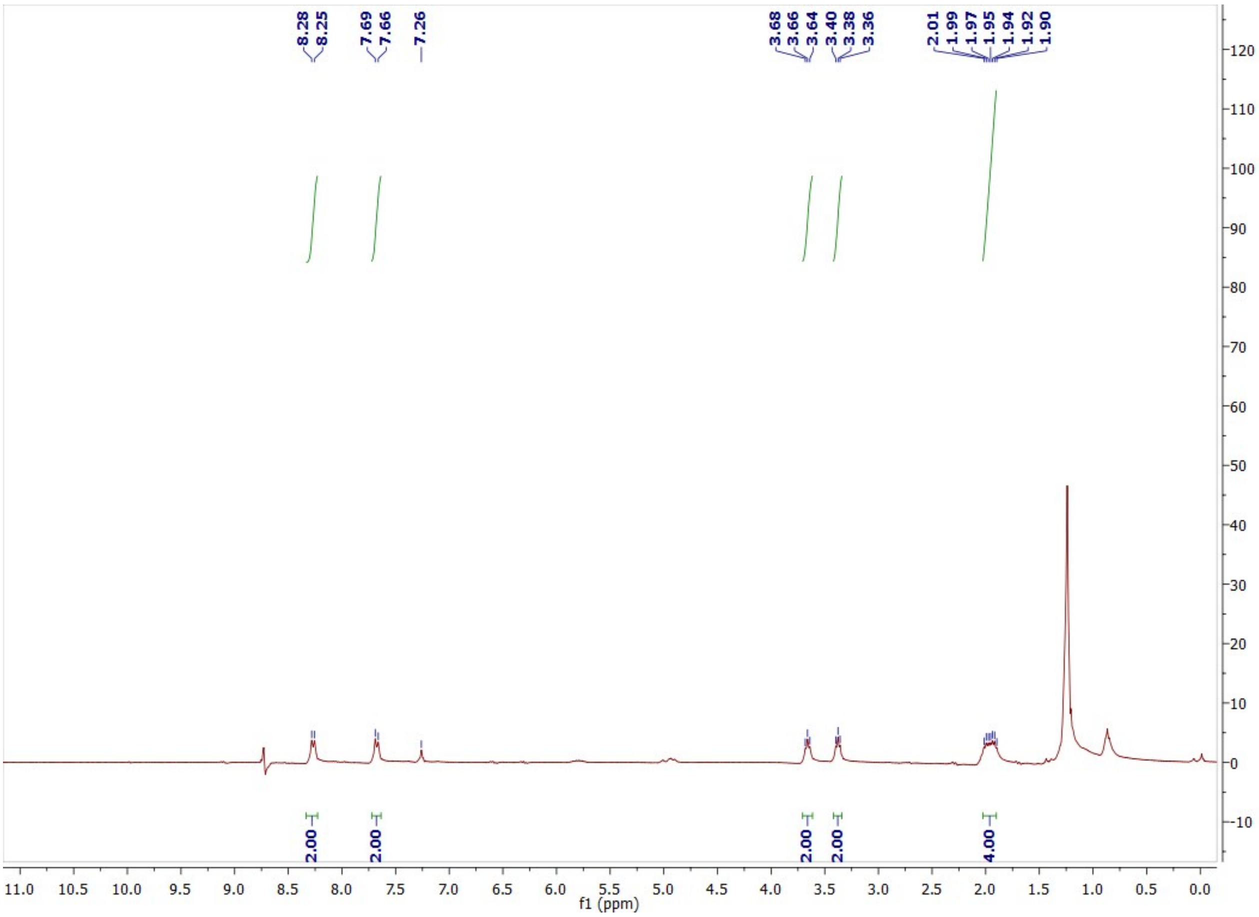

Fig. S25 ^{1}H NMR (300 MHz, $CDCl_3$) spectrum of **5a**.

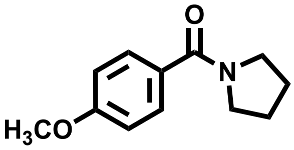

5b

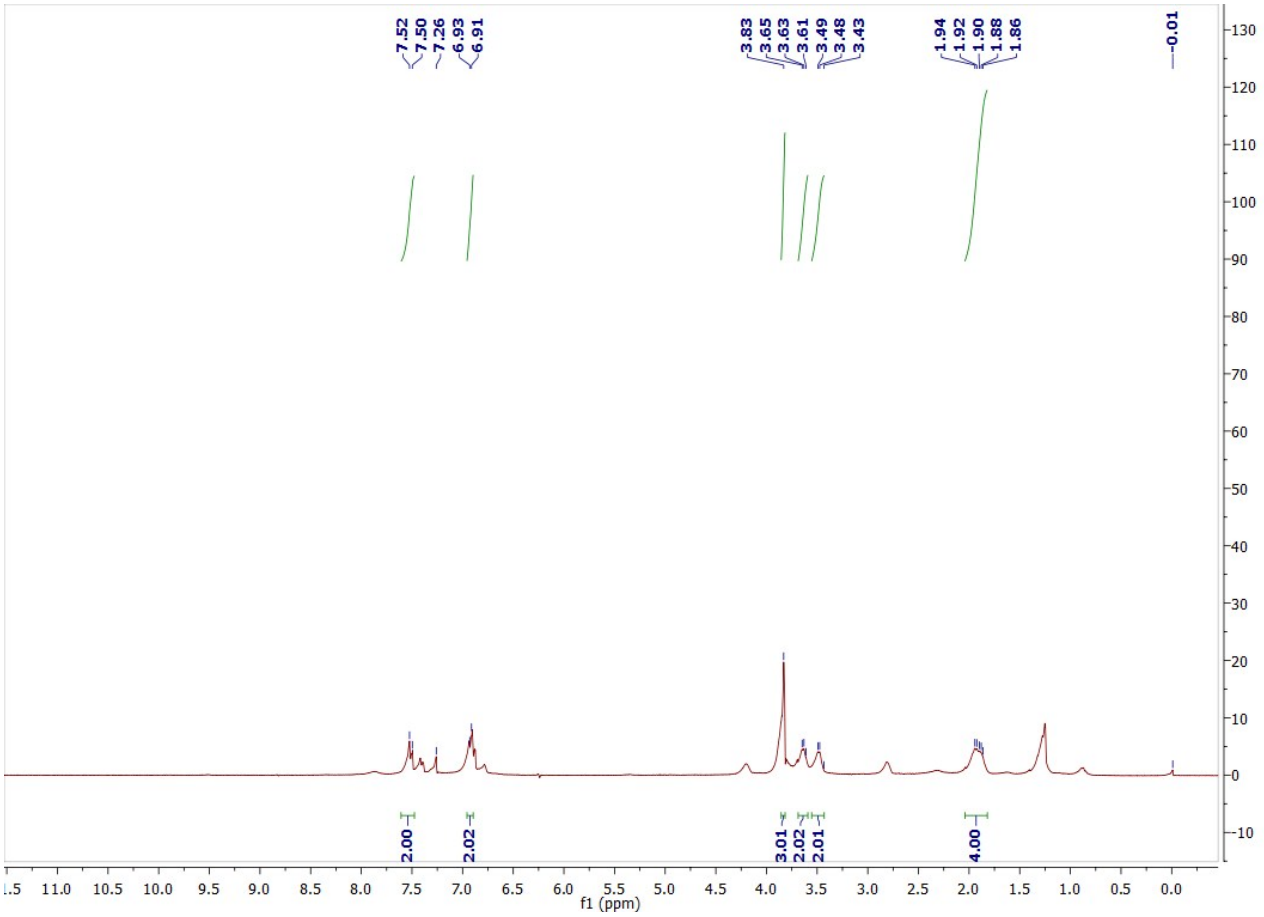

Fig. S26 ^{1}H NMR (300 MHz, $CDCl_3$) spectrum of **5b**.

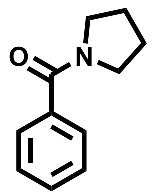

5c

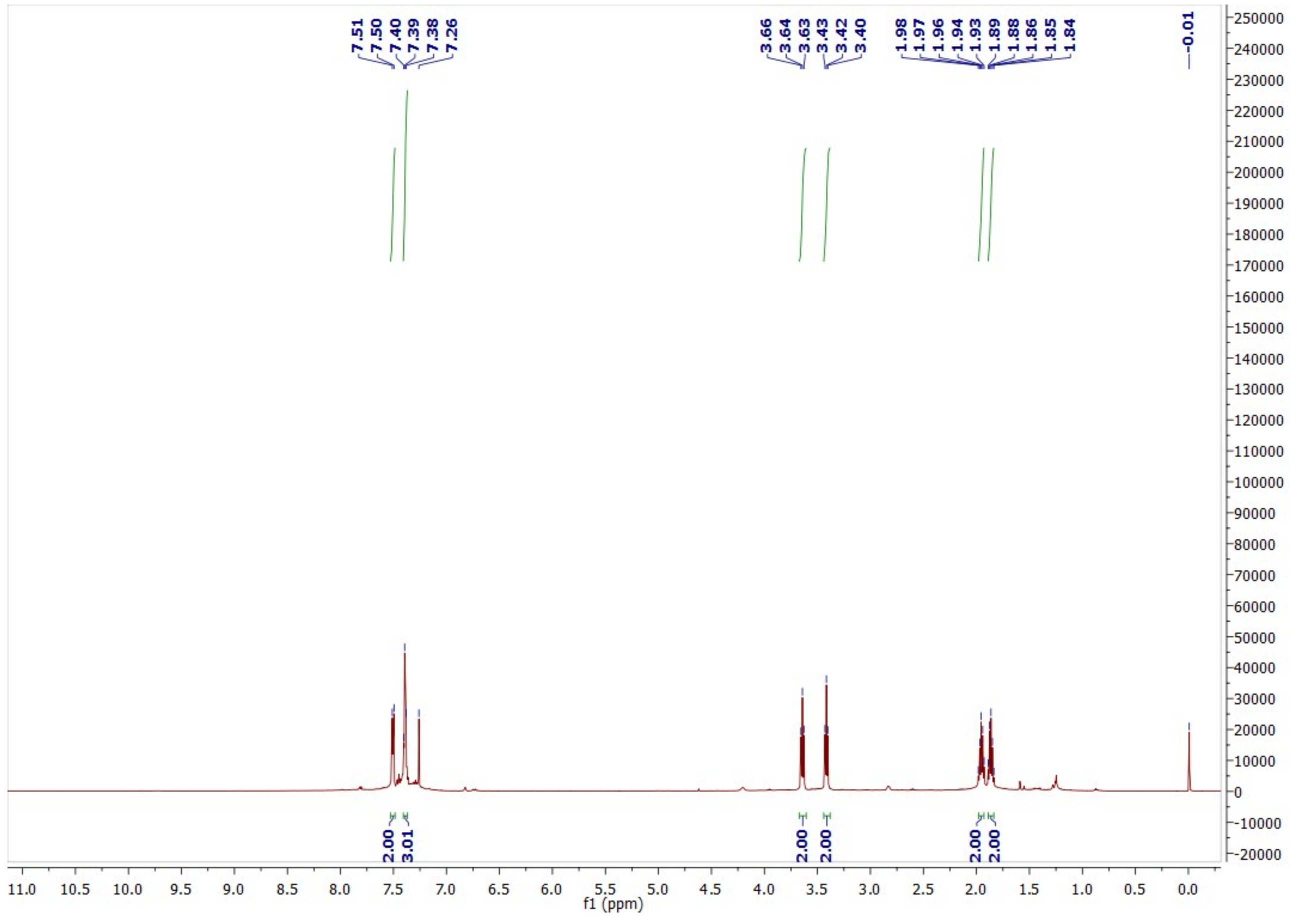

Fig. S27 ^{1}H NMR (500 MHz, $CDCl_3$) spectrum of **5c**.

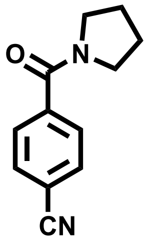

5d

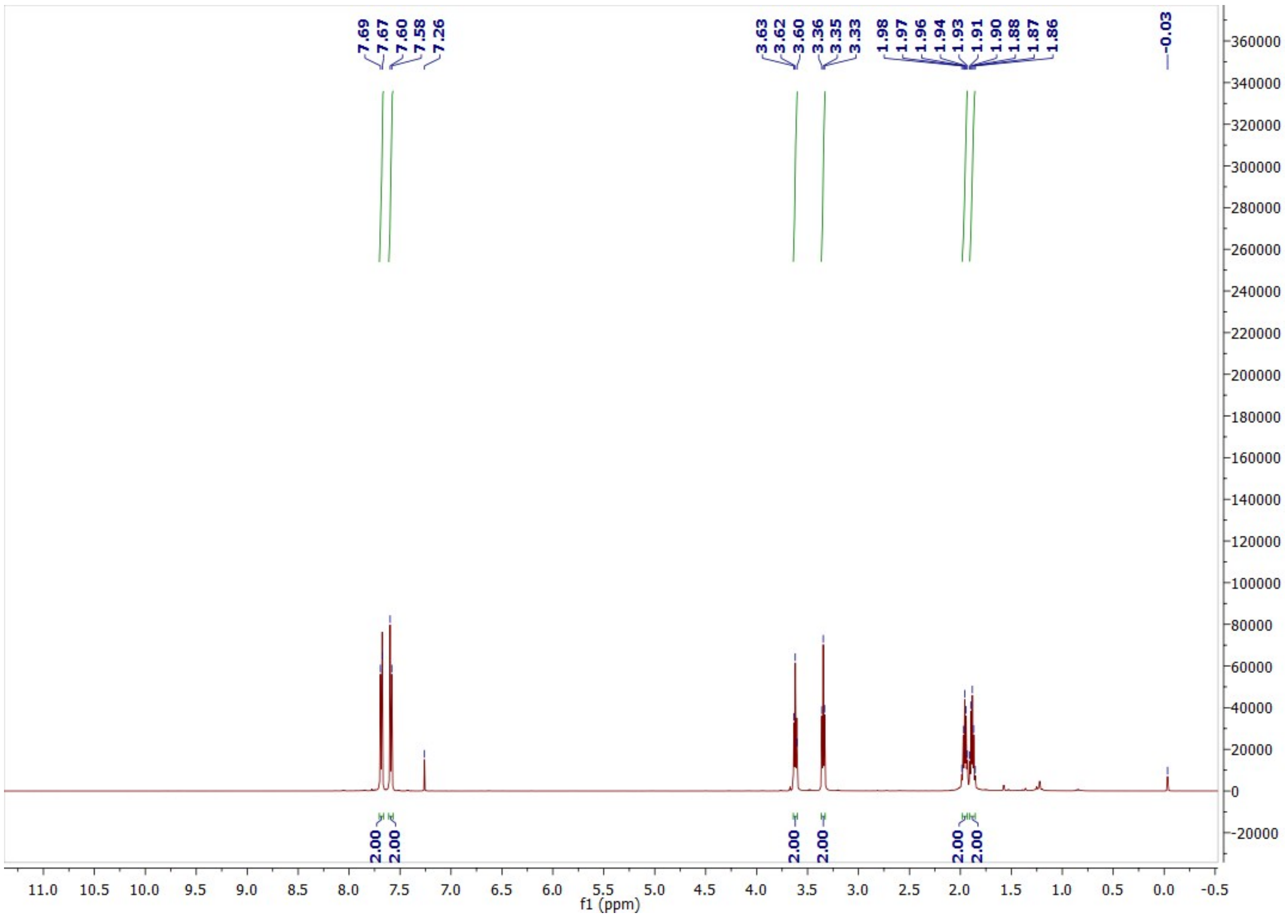

Fig. S28 ^{1}H NMR (500 MHz, $CDCl_3$) spectrum of **5d**.

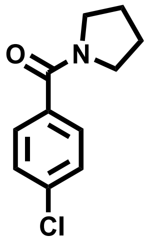

5e

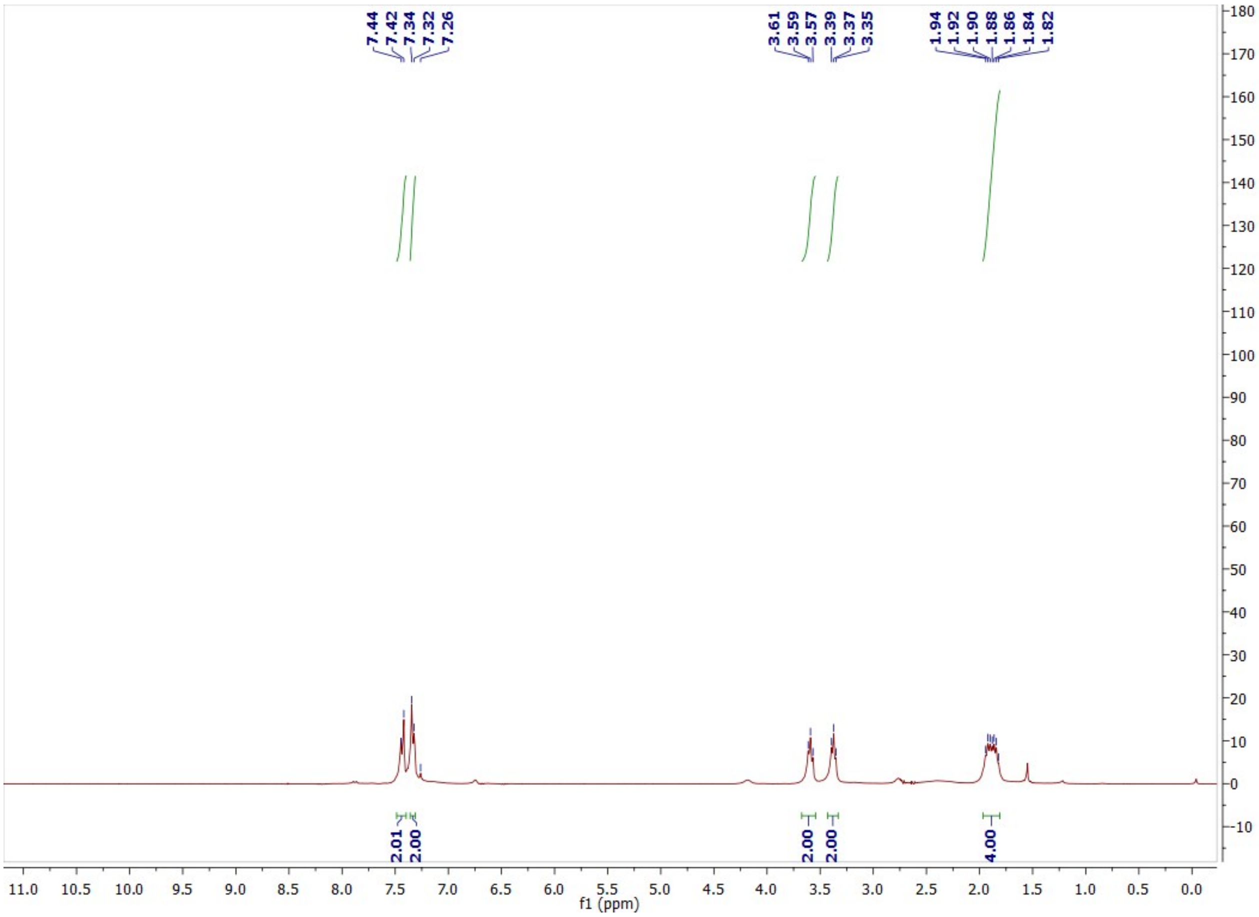

Fig. S29 ^{1}H NMR (300 MHz, $CDCl_3$) spectrum of **5e**.

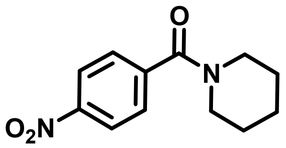

7a

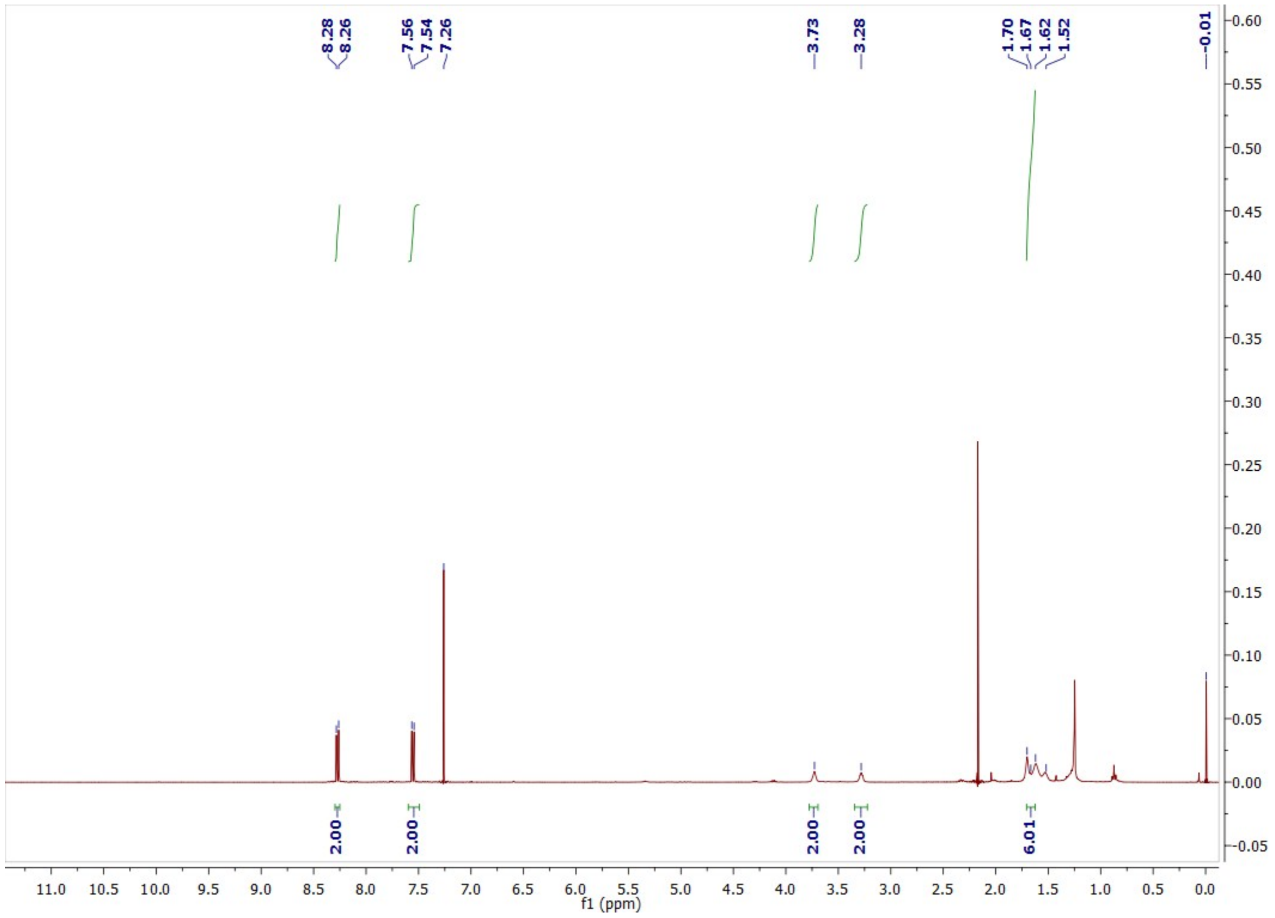

Fig. S30 ^{1}H NMR (400 MHz, $CDCl_3$) spectrum of **7a**.

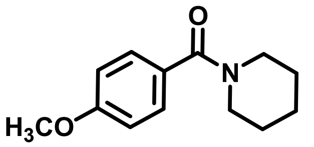

7b

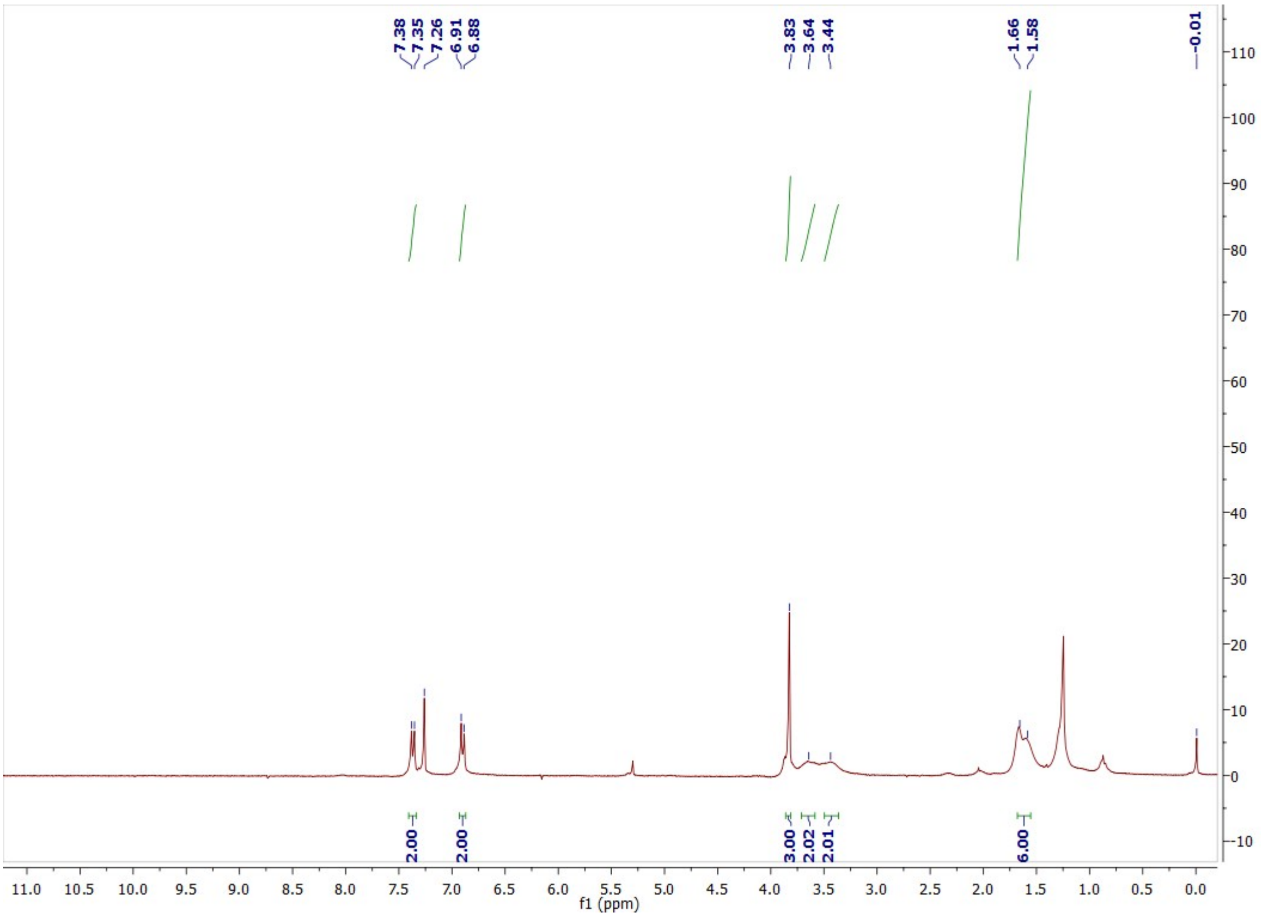

Fig. S31 ^{1}H NMR (300 MHz, $CDCl_3$) spectrum of **7b**.

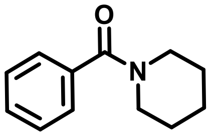

7c

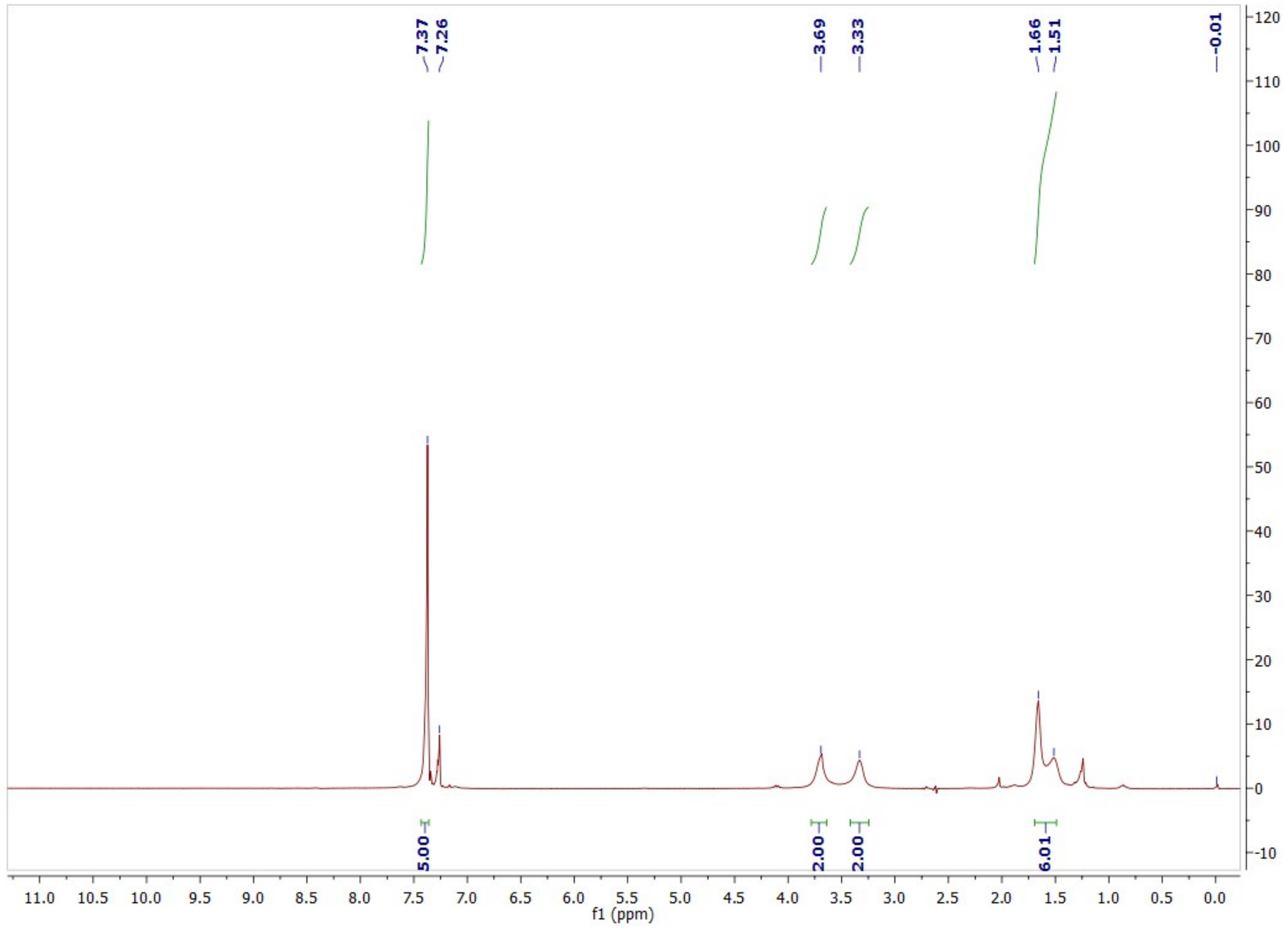

Fig. S32 ^{1}H NMR (300 MHz, $CDCl_3$) spectrum of **7c**.

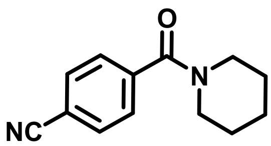

7d

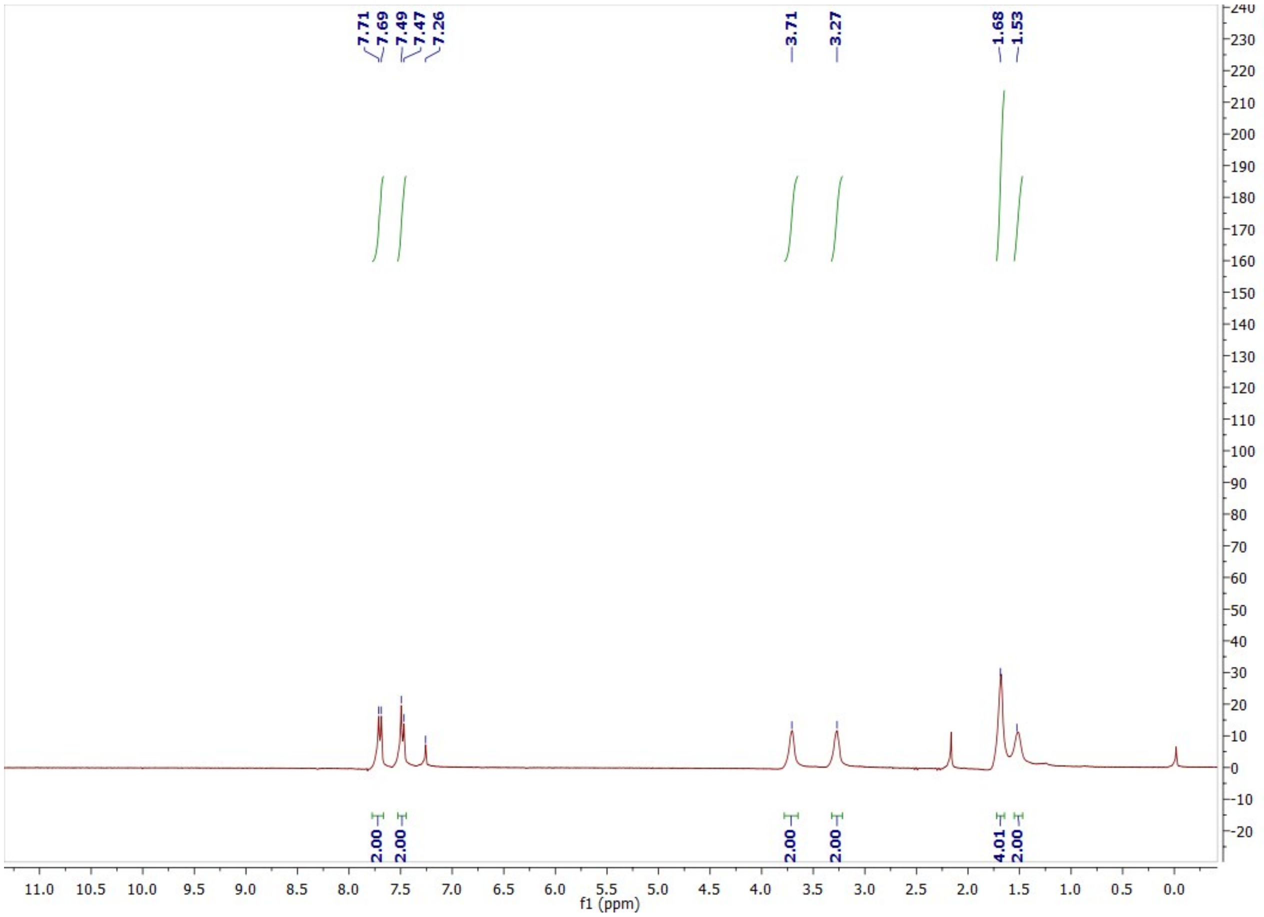

Fig. S33 ^{1}H NMR (300 MHz, $CDCl_3$) spectrum of **7d**.

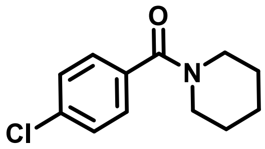

7e

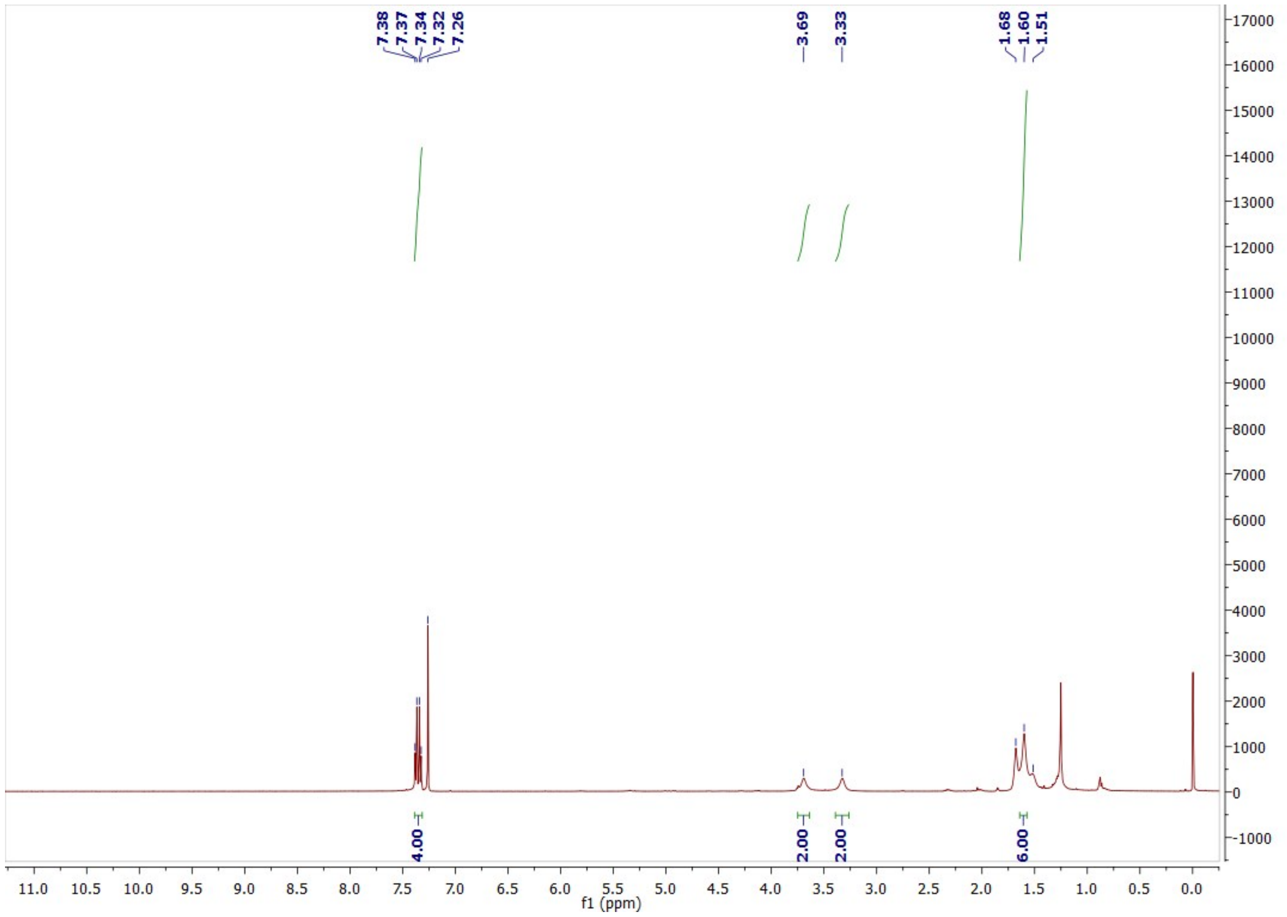

Fig. S34 ¹H NMR (500 MHz, $CDCl_3$) spectrum of **7e**.

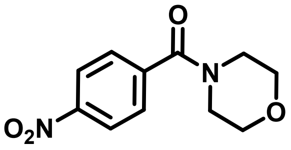

8a

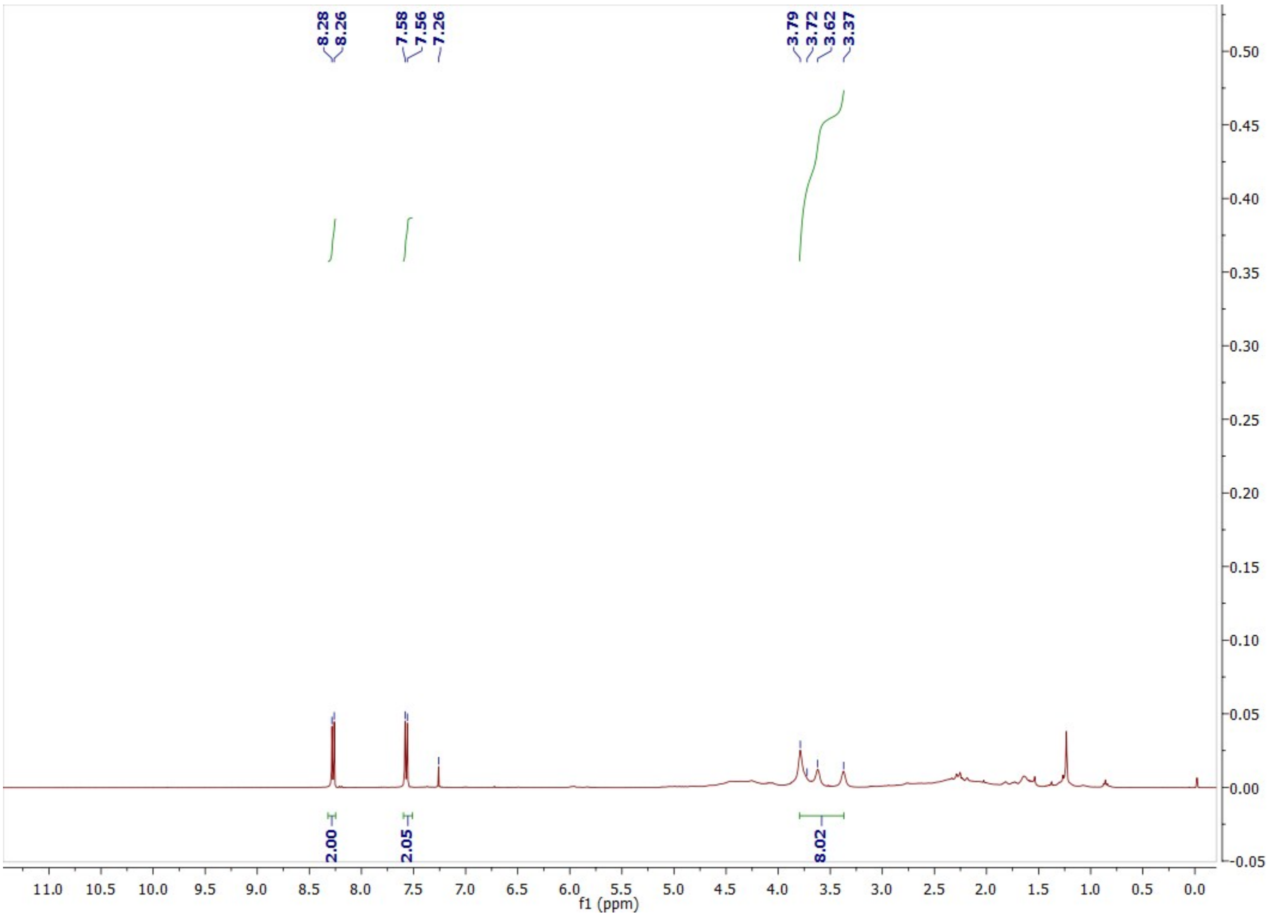

Fig. S35 ^{1}H NMR (400 MHz, $CDCl_3$) spectrum of **8a**.

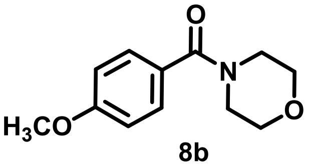

8b

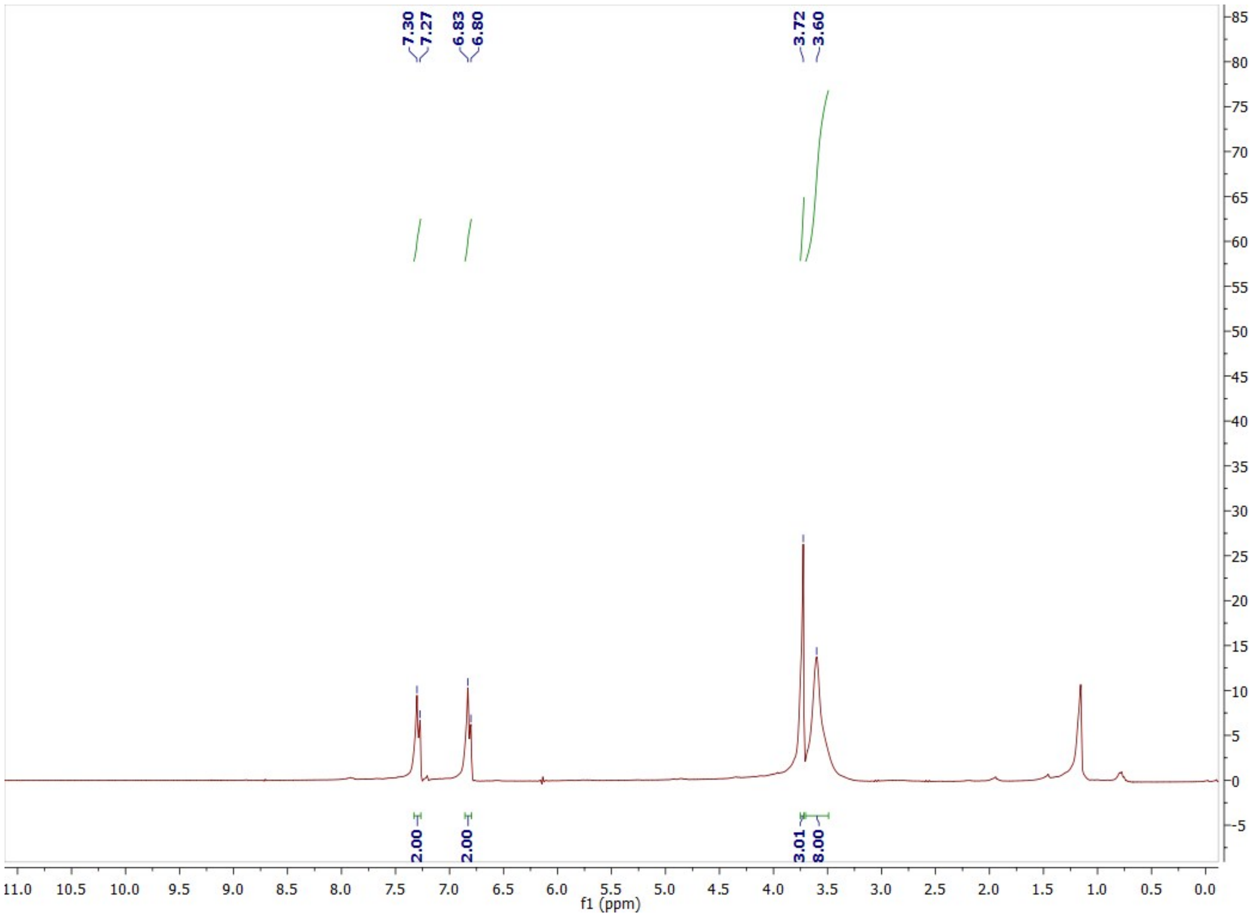

Fig. S36 ^{1}H NMR (300 MHz, $CDCl_3$) spectrum of **8b**.

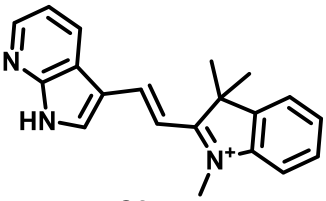

C2

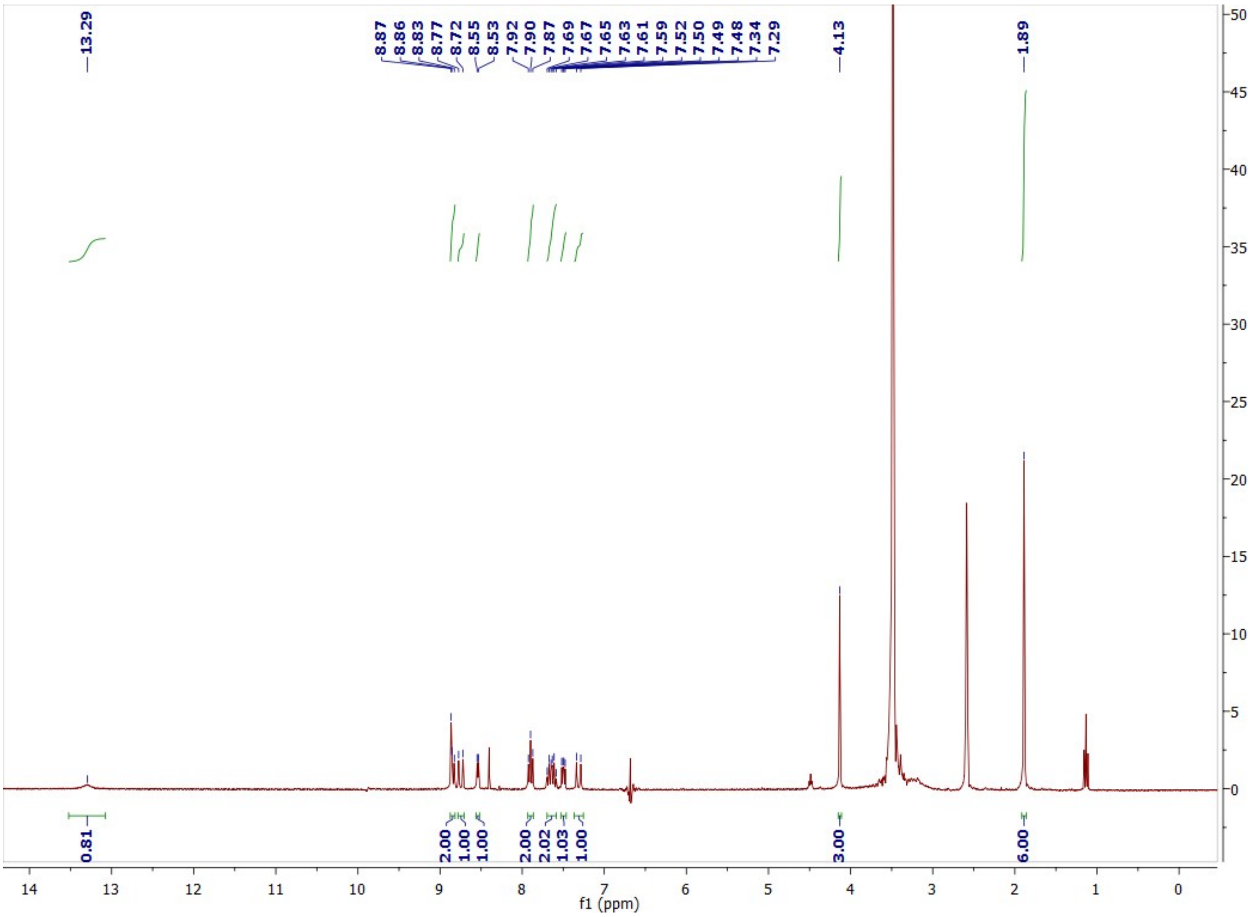

Fig. S37 ^{1}H NMR (300 MHz, $CDCl_3$) spectrum of **C2**.

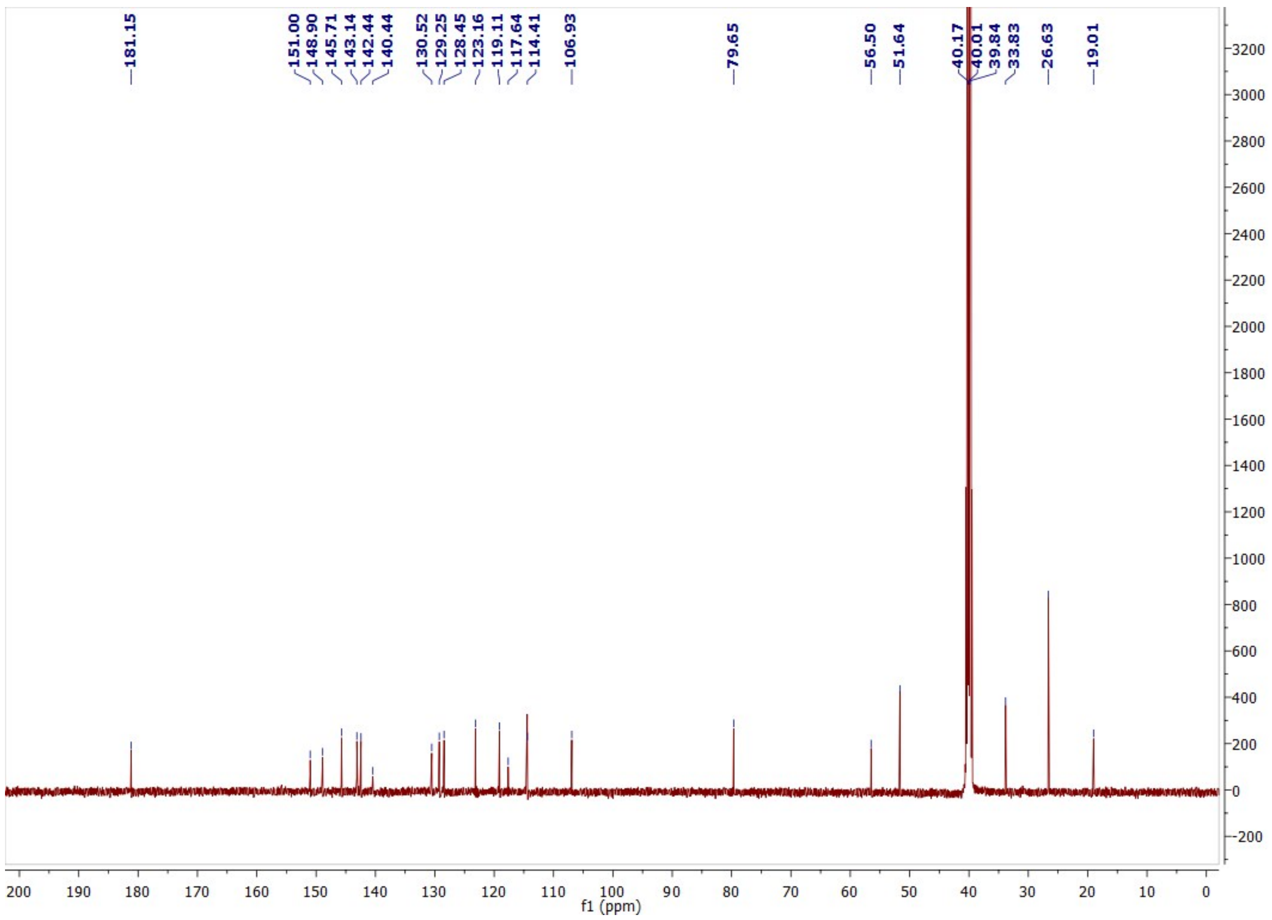

Fig. S38 ^{13}C NMR (500 MHz, $CDCl_3$) spectrum of **C2**.

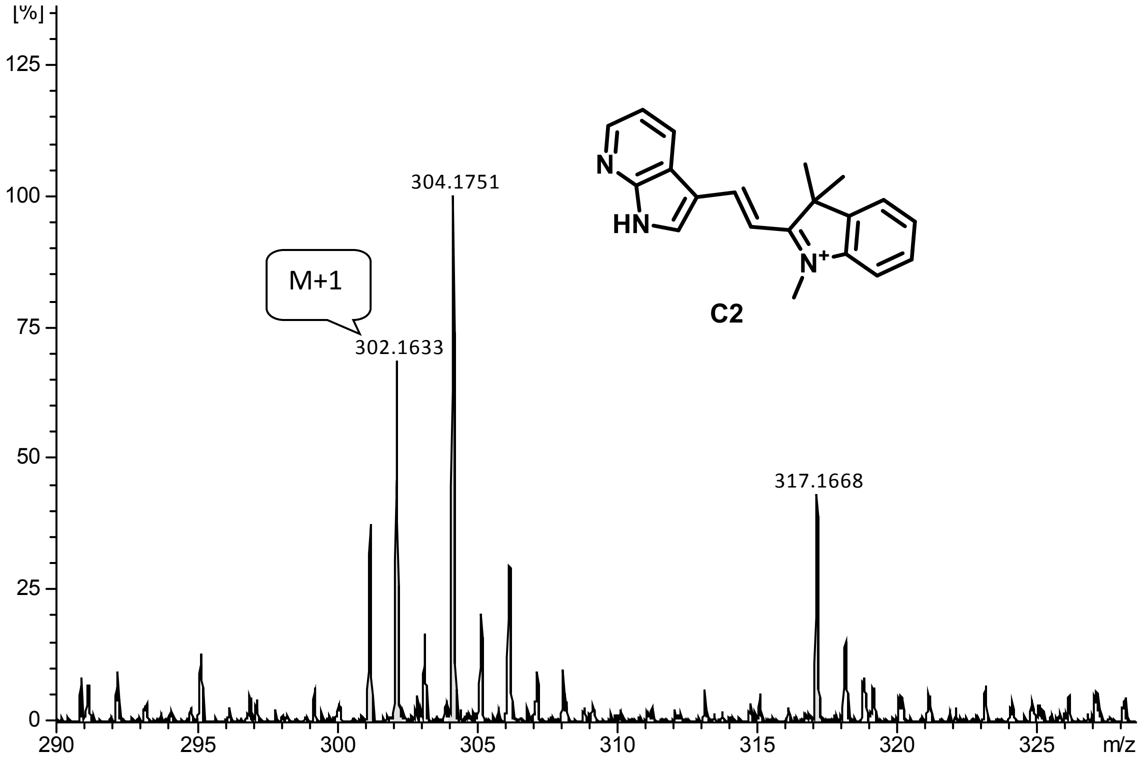

Fig. S39 Mass spectrum of **C2**.

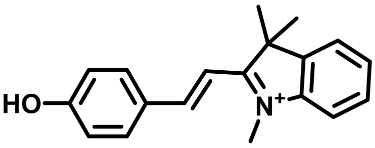

C3

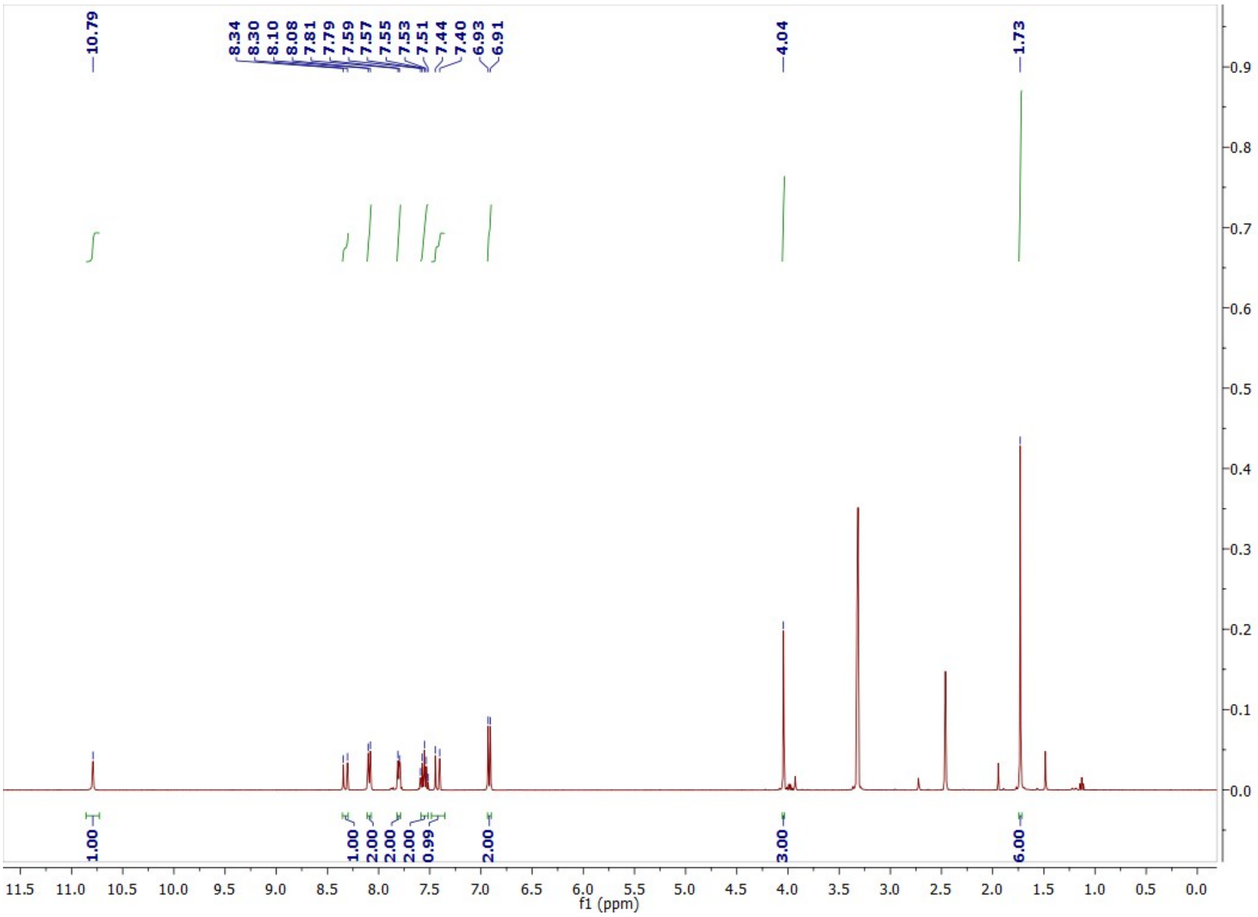

Fig. S40 ^{1}H NMR (400 MHz, $CDCl_3$) spectrum of **C3**.

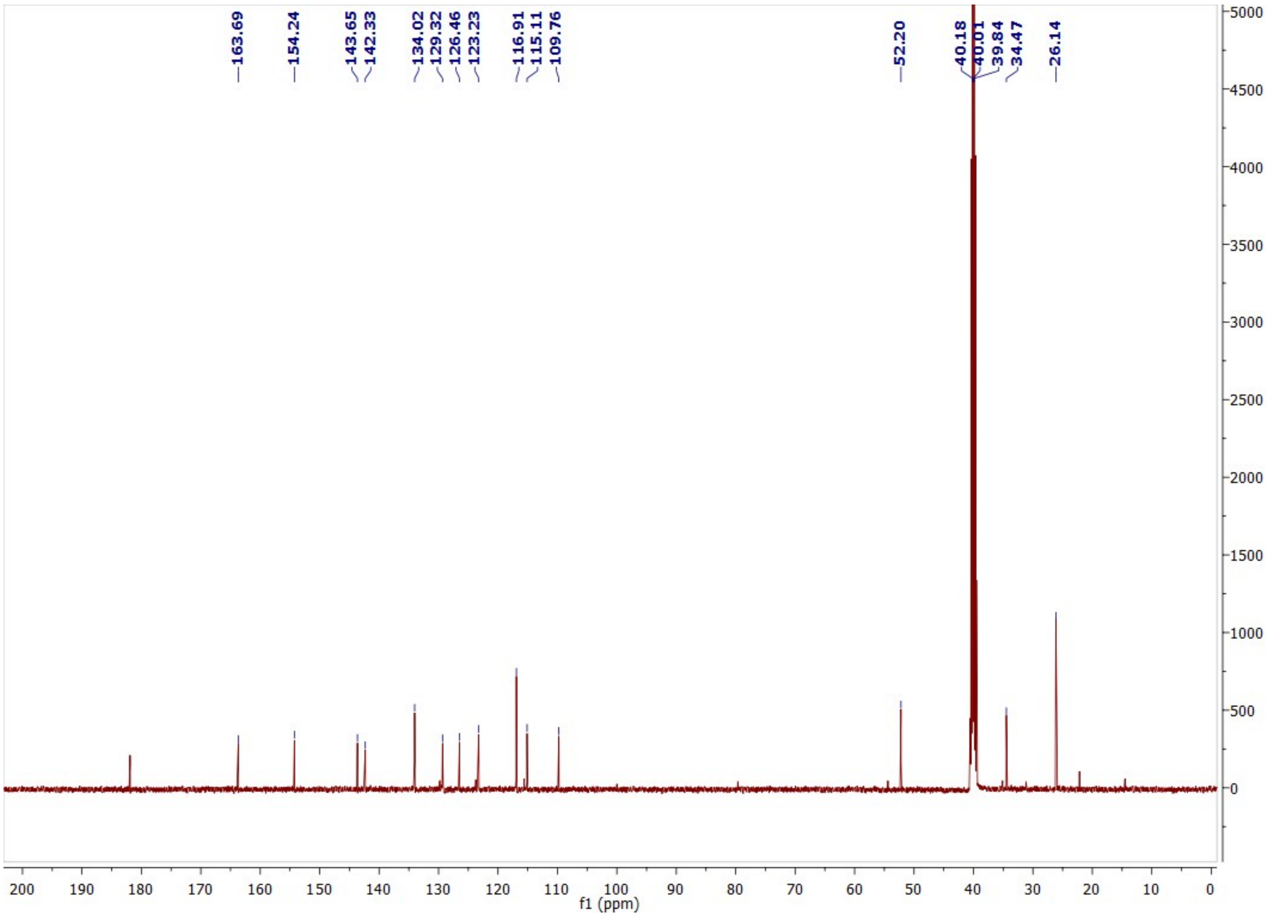

Fig. S41 ^{13}C NMR (500 MHz, DMSO-d_6) spectrum of **C3**.

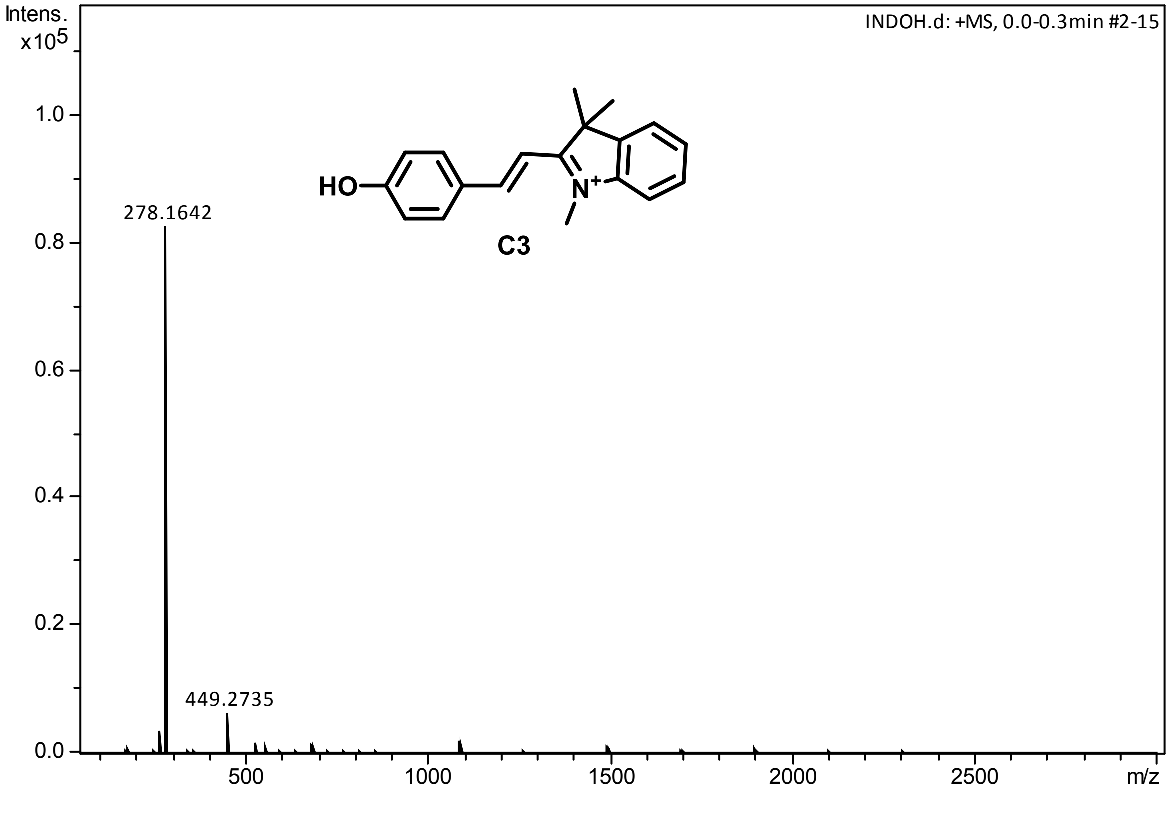

Fig. S42 Mass spectrum of **C3**.

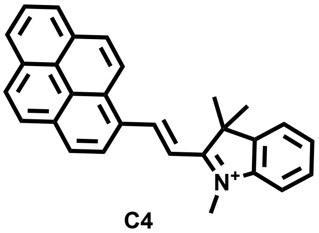

C4

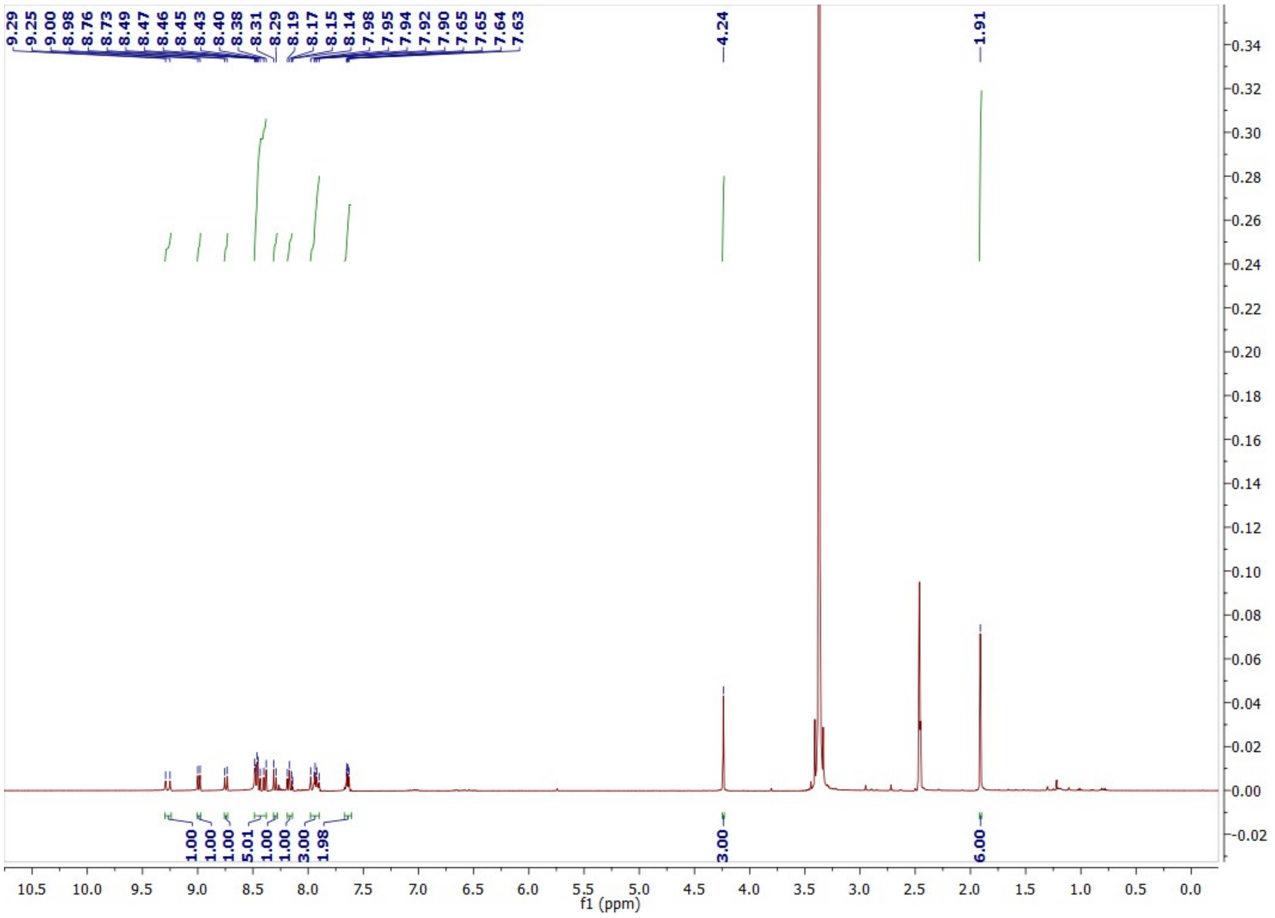

Fig. S43 ^{1}H NMR (400 MHz, DMSO-d_6) spectrum of **C4**.

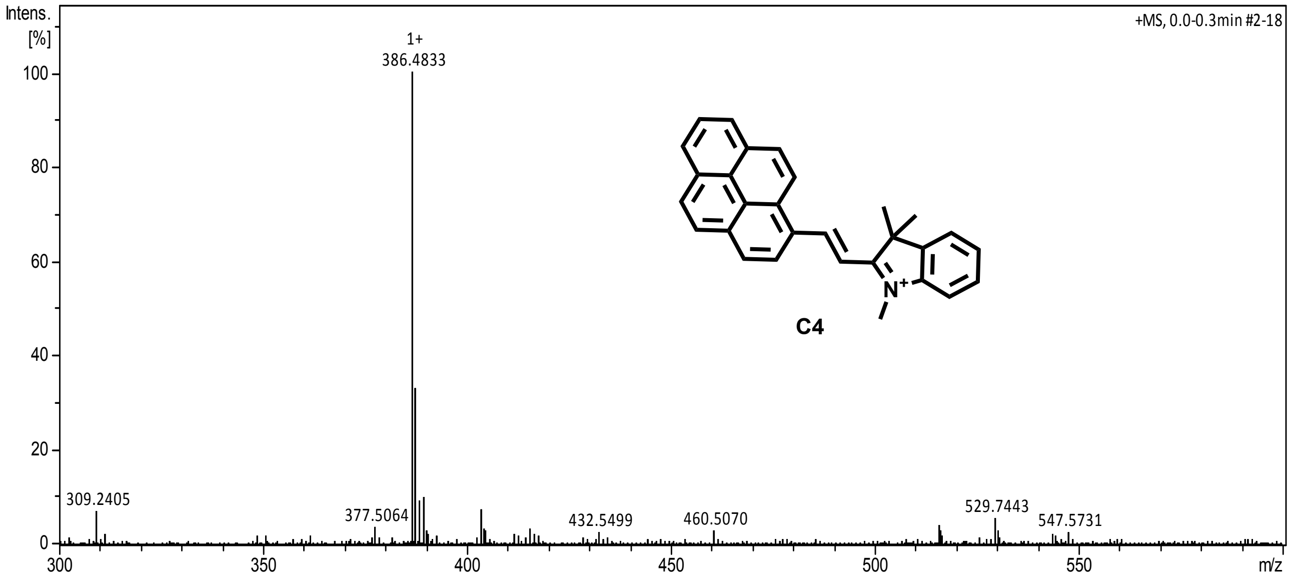

Fig. S44 Mass spectrum of **C4**.
